# Effects of pre-mixed gas composition on combustion characteristics of micro pilot dual fuel (MPDF) in a heavy-duty dual fuel engine

**DOI:** 10.1038/s41598-022-11958-3

**Published:** 2022-05-12

**Authors:** Minhoo Choi, Sungwook Park

**Affiliations:** School of Mechanical Engineering, Hanyang University, 222 Wangsimni-ro, Seongdong-gu, Seoul, 04763 Republic of Korea

**Keywords:** Mechanical engineering, Fossil fuels

## Abstract

The effects of premixed gas compositions on the combustion characteristics in micro pilot duel fuel (MPDF) conditions were investigated. Propane, hydrogen, and carbon dioxide gases were added to methane gas, and engine experiments were conducted under various premixed gas compositions. A single-cylinder heavy-duty engine with a combustion chamber volume of 1100 mm^3^ and compression ratio of 17.0 was used. A 55 kW DC dynamometer was used to operate the single-cylinder dual-fuel engine at a constant engine speed. At high propane mixture ratios, knocking combustion occurred, accompanied by intense engine vibrations, owing to the low octane number of propane. Knocking combustion led to an increase in the combustion variation and ringing intensity (which represents the knocking combustion intensity). In contrast, at high ratios of hydrogen, which has a high octane number, knocking combustion was suppressed, and the speed of combustion was lower than that in the case of high propane mixture ratios. The optimum conditions corresponded to a ringing intensity of 3–5 MW/m^2^. The addition of even a small amount of propane gas enhanced the engine performances in misfiring conditions. In contrast, a considerable amount of hydrogen gas was required to prevent abnormal combustion because of the low density of hydrogen gas. The presence of carbon dioxide effectively stabilized MPDF combustion by suppressing knocking combustion.

## Introduction

Emissions from diesel vehicles have been a key global issue for several decades. In particular, heavy-duty diesel engines, which have high compression ratios and operating loads, emit considerable amounts of harmful components. Moreover, the diffusion combustion occurring in diesel engines is the primary source of nitrogen oxide (NOx) and particulate matter (PM) emissions. NOx and PM are generated in different regimes of diffusion combustion. NOx is produced in regions of local stoichiometric equivalence in which the combustion temperature exceeds 2300 K, whereas PM formation occurs in a rich equivalence ratio region in which the combustion temperature is low^1–3^. The significant PM emissions are also attributable to the characteristics of diesel fuel, such as the presence of multiple C–C bonds and low H/C ratios^4,5^. The use of diesel fuel in compressed ignition (CI) engines thus involves considerable emissions of NOx and PM. Several researchers have focused on the use of alternative fuels for decreasing the NOx and PM emissions. The diesel mixture ratio can be decreased by implementing dual-fuel combustion, in which alternative fuel is used as the main source of power and diesel or biofuel in low proportions is used as an ignitor^6–8^. Among alternative fuels, methanol, natural gas, and methane (CH_4_) are typically used for dual-fuel combustion. The effects of the fuel mixture ratio on the amount of emissions in dual-fuel combustion has been studied^9–11^. The amount of NOx emissions was noted to be proportional to the diesel mixture ratio in dual-fuel combustion. Choi et al.^11^ indicated that micro pilot dual fuel (MPDF) combustion, which involves an extremely small amount of diesel, can simultaneously decrease NOx and PM emissions by changing the diffusion combustion pertaining to diesel to premixed combustion. Moreover, MPDF combustion corresponds to reduced CO_2_ emissions because of the low number of carbons^11,12^. Several researchers have also reported on reducing emissions by using alternative fuels for dual-fuel combustion^13–23^.

Although MPDF combustion can decrease the amount of emissions, abnormal combustion such as misfiring and knocking combustion may occur, which can increase the combustion variation and deteriorate the thermal efficiency^24–26^. The MPDF combustion forms are affected by the engine operating conditions and premixed gas compositions. The engine operating conditions are primarily influenced by the intake air temperature, equivalence ratio, and diesel injection timing^27–29^. Moreover, under the same engine operating conditions, misfiring and knocking combustion may occur depending on the premixed gas compositions. The premixed gas compositions can be optimized to prevent abnormal combustion forms in MPDF conditions, and thus, several researchers have focused on investigating the effects of premixed gas compositions on MPDF combustion. Many variants of CH_4_ gas have been used to conduct engine experiments. Notably, the additive gases can be categorized into flammable and non-flammable inert gases. Moreover, flammable gases can be classified into hydrocarbon and non-hydrocarbon based gases. In this study, propane (C_3_H_8_), hydrogen (H_2_), and carbon dioxide (CO_2_) gases were selected as representatives of hydrocarbon based, non-hydrocarbon flammable, and non-flammable inert gases, respectively.

Hydrocarbon gases can lead to knocking combustion owing to their low octane numbers (OCNs)^30^. Polk et al.^31^ investigated the effects of C_3_H_8_ on the combustion and emission characteristics of a turbocharged direct-injection diesel engine. The authors reported that C_3_H_8_ significantly advanced the mass fraction burned (MFB) crank angle (CA) 50 and increased the NOx emissions under high engine loads. Gibson et al.^32^ compared the performance and emission characteristics of a turbocharged direct injection dual-fuel engine using CH_4_ and C_3_H_8_ gases. The NOx and PM emissions were noted to be significantly reduced by dual-fuel combustion. Moreover, C_3_H_8_ exhibits a higher brake thermal efficiency than CH_4_ owing to a high reactivity. However, under high engine loads, end-gas knocking combustion easily occurs when using C_3_H_8_.

H_2_, which has a high flame propagation speed, promotes combustion^33^. However, H_2_ suppresses knocking combustion. Topinka et al.^34^ noted that the presence of H_2_ can decelerate ignition reactions owing to its high OCN. Liu et al.^35^ analyzed the effects of the volume ratio of H_2_ on the combustion characteristics of an optical single-cylinder dual-fuel engine. At high ratios of H_2_, the peak cylinder pressure significantly increased, and the combustion phase was advanced. Sanli et al.^36^ investigated the effects of CH_4_ and H_2_ mixture ratio on combustion and emissions characteristics. According to their research, increasing H_2_ mixture ratio reduced the ignition delay, advancing the combustion phasing. Moreover, the H_2_ effectively decreased the incomplete combustion materials and CO_2_ emissions, but the NOx emissions were inevitably.

Unlike the flammable gases, CO_2_ leads to low-temperature combustion, which decreases the combustion rate and prevents knocking. CO_2_, which has a large heat capacity, can decrease the combustion temperature^37,38^. In other words, increasing the CO_2_ gas ratio in premixed gas fuel has the same effect as that of applying exhaust gas recirculation (EGR) technologies. Tomita et al.^27^ investigated the effects of the EGR rate on the combustion and emission characteristics of a supercharged dual-fuel engine with natural gas and diesel fuel. Under high EGR rate conditions, the combustion phasing was retarded, and low NOx emissions were generated. However, the presence of large proportions of CO_2_ in premixed gas may lead to misfiring, which deteriorates the engine performance and promotes the incomplete combustion of gases.

Although several preliminary studies have been conducted to analyze the effects of premixed gas compositions on MPDF combustion, comprehensive research on classifying the MPDF combustion forms and optimizing the premixed gas compositions for various engine operating conditions remains limited. Therefore, in this study, the effects of premixed gas compositions on MPDF combustion characteristics was investigated by conducting engine experiments for various mixture ratios of C_3_H_8_, H_2_, and CO_2_ gases.

## Experiment details

### Setup

**Figure 1:**
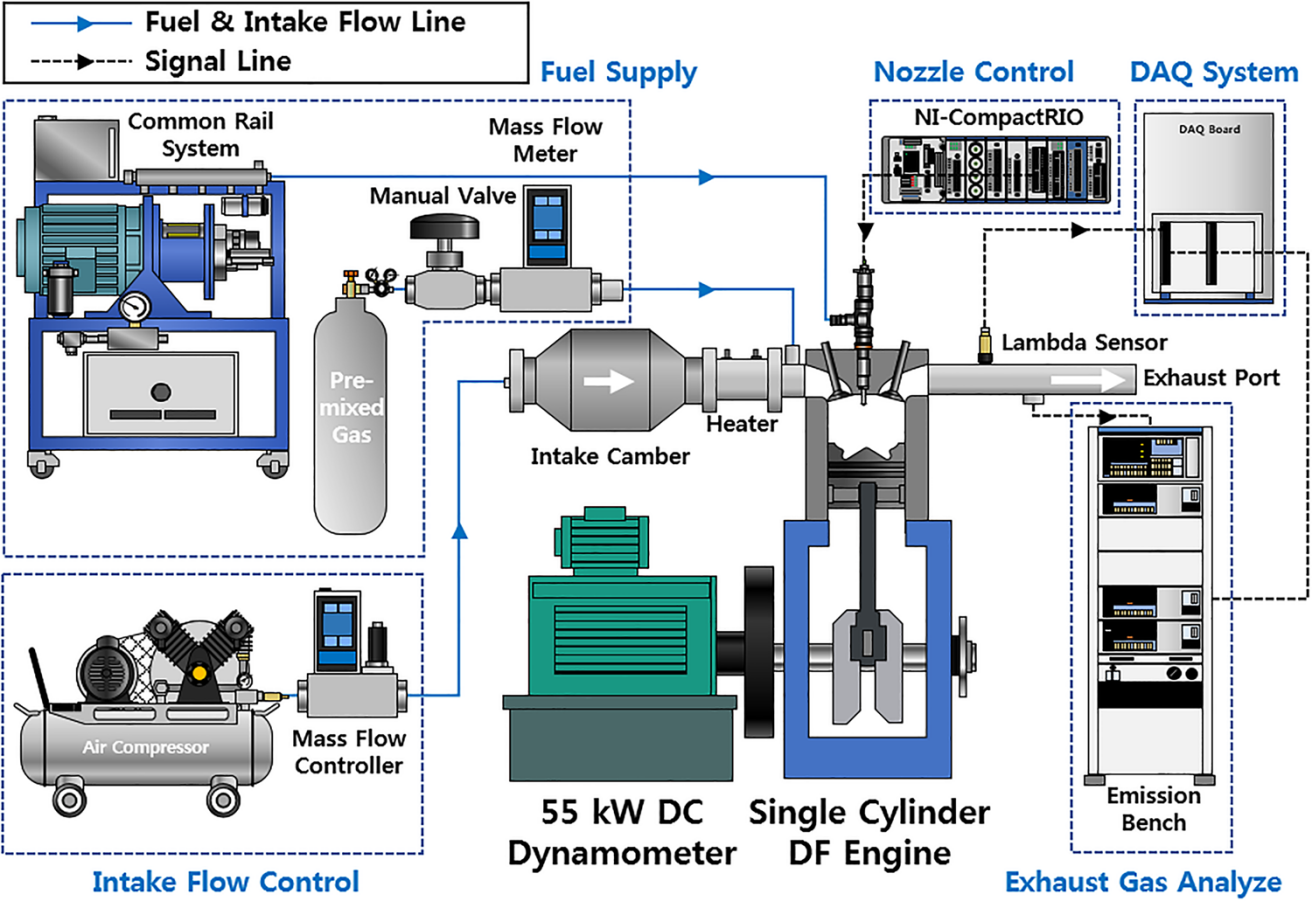
Schematic of apparatus used in the experiment.

Experiments were conducted in a single-cylinder heavy-duty engine with a combustion chamber volume of 1100 mm^3^ and compression ratio of 17.0. Table 1 presents the specifications of the single-cylinder dual-fuel engine. Figure 1 schematically illustrates the experiment setup. A 55 kW DC dynamometer was used to operate the single-cylinder dual-fuel engine at a constant engine speed. In addition, a mass flow controller (MFC) and mass flow meter (MFM) were used to accurately supply the air and premixed gas, respectively. The intake air was automatically controlled, and the premixed gas flow rate was controlled using a manual valve to ensure safety. Air was supplied to the intake chamber to decrease the fluctuation of the intake pressure. After the intake chamber, a heater was installed to maintain the intake air temperature. The premixed gas was mixed with the intake air from the intake port. Unlike the air and premixed gas, micro pilot (MP) diesel was directly injected into the combustion chamber. A common rail system was used to maintain the diesel injection pressure, and the MP injection timing and duration were controlled using an NI-CompactRIO system. The exhaust gas analyzer (HORIBA-MEXA 9,100D) was connected to the exhaust port, and it detected the emission concentrations of NOx, CO, and O_2_ gases. Experimental data were acquired using the NI-DAQboard and LabVIEW program. Hundred cycles of the acquired data were averaged to minimize the variation in the experiment results.

**Table 1:** Specifications of single-cylinder dual-fuel engine.

Parameter	Value
Bore (mm)	107
Stroke (mm)	126
Connecting rod (mm)	200
Displacement volume (mm^3^)	1100
Compression ratio	17.0
Number of injector nozzle holes	7
Nozzle hole diameter (mm)	0.174

### Experiment conditions

Table 2 lists the reference experiment conditions. Because the engine experiments were conducted under a high engine operating load, a low engine speed was selected, and the intake air pressure was increased to 2.1 bar. To implement MPDF combustion, the MP injection quantity was selected as the minimum amount that did not result in misfiring. The MP injection pressure was selected as 500 bar, and the MP injection duration, which determines the diesel injection quantity, was derived through preliminary experiments. In the preliminary experiments, the MP injection duration was varied from 0.23 ms to 0.40 ms, and the combustion variation was compared. High combustion variations were observed at MP injection durations of 0.23 ms and 0.27 ms, attributable to the low ignition intensity. The findings highlighted that the MP injection duration must be more than 0.30 ms to prevent high combustion variations. Therefore, the MP injection duration of 0.30 ms, for which the lower heating value (LHV) of diesel was 45.8 J/stroke, was selected as the reference condition. For all experimental cases, the MP injection pressure and duration were maintained as 500 bar and 0.30 ms, respectively.

**Table 2:** Reference experiment conditions.

Parameter	Value
Engine speed (RPM)	900
Intake air pressure (bar)	2.1
MP injection pressure (bar)	500
MP injection duration (ms)	0.3
LHV of diesel fuel (J/stroke)	45.8
MP injection timing (bTDC)	27.0
Lambda (1/equivalence ratio)	2.2
LHV of CH_4_ (J/stroke)	2976.3
Intake temperature (°C)	35
Coolant and oil temperatures (°C)	80

Because the portion of MP injection quantity was extremely small compared to that of the premixed gas, only the premixed gas was considered for calculating the equivalence ratio. The premixed gas flow rate was calculated based on the lambda value. For the reference condition corresponding to pure CH_4_ and lambda 2.2, the LHV of premixed gas was 2,976.3 J/stroke. Because the engine experiments were performed under high engine loads, the MP injection timing approaching the top dead center (TDC) was expected to lead to the auto-ignition of premixed gas, resulting in knocking combustion. To prevent knocking combustion, the MP injection timing was required to occur before the TDC. Therefore, the MP injection timing before top dead center (bTDC) at 27°, a low frequency of auto-ignition, was selected as the reference condition. The intake air temperature was maintained as low as possible. The temperature of the coolant and oil was 80.0 °C.

The engine experiments were conducted using various premixed gas compositions. CH_4_ gas was selected as the main component of the premixed gas fuel and blended with other gases (C_3_H_8_, H_2_, and CO_2_). Table 3 presents the specifications of premixed gas compositions^39^. C_3_H_8_ was selected as a representative hydrocarbon-based gas. C_3_H_8_ has a lower OCN and higher LHV than CH_4_. Because knocking combustion can easily occur at high C_3_H_8_ mixture ratios, the percentage of C_3_H_8_ was varied to 3%, 5%, 7%, and 10% of the fuel. H_2_ was used as a non-hydrocarbon flammable gas. Unlike C_3_H_8_, H_2_ has a high OCN and can suppress knocking combustion. Therefore, the ratio of H_2_ was larger than that of C_3_H_8_ because of the low possibility of knocking combustion. In addition, because of the low density and LHV per unit volume (MJ/m^3^) of H_2_, a large amount of H_2_ was required to achieve the reference equivalence ratio. The H_2_ mixture ratios were 10%, 20%, and 30%. CO_2_ was selected as an inert gas that does not participate in the combustion reaction, and thus, the LHV, stoichiometric air–fuel ratio and OCN of CO_2_ are not presented in Table 3. As in the case of H_2_, the CO_2_ mixture ratios were 10%, 20%, and 30%.

**Table 3:** Specification of premixed gas compositions at standard states^39^.

Parameters		Premixed gas compositions			
		CH_4_	C_3_H_8_	H_2_	CO_2_
LHV	MJ/kg	55.60	49.60	143.00	N/A
	MJ/m^3^	37.80	83.60	10.70	N/A
AFR (mass)		17.16	15.60	34.32	N/A
OCN		125	105	130	N/A
Auto-ignition temperature (°C)		540	490	585	N/A
Mixture ratio (%)		100 – Additive Gas	3, 5, 7, 10	10, 20, 30	10, 20, 30

Because this study focused on the effects of premixed gas compositions on the MPDF combustion characteristics, various parameters, such as the intake air pressure, intake air temperature, MP injection parameters, and lambda value were maintained constant, and only the premixed gas compositions were varied. In general, the combustion intensity and rate cannot be compared considering the rate of heat release and accumulated heat release because the total LHV varies with the premixed gas composition. Therefore, the normalized rate of heat release and MFB were calculated to determine the rate of combustion, as follows:

1 $$Accumulated\; Heat\; Release\;_{\theta } \left( J \right) = \mathop \smallint \limits_{Intake\; Valve\; Close}^{\theta } Rate\; of\; Heat\; Release\; d\theta$$

2 $$Mass\; Fraction \;Burned\; \left( \% \right) = \frac{{Accumulated\; Heat \;Release\;_{\theta } }}{{Accumulated \;Heat \;Release\;_{Exhaust \;Valve \;Open} }}$$

3 $$Normalized\; Rate \;of \;Heat \;Release \;\left( {\% /deg} \right) = \frac{Rate \;of \;Heat \;Release}{{Accumulated \;Heat \;Release\;_{Exhaust \;Valve \;Open} }}$$

**Figure 2:**
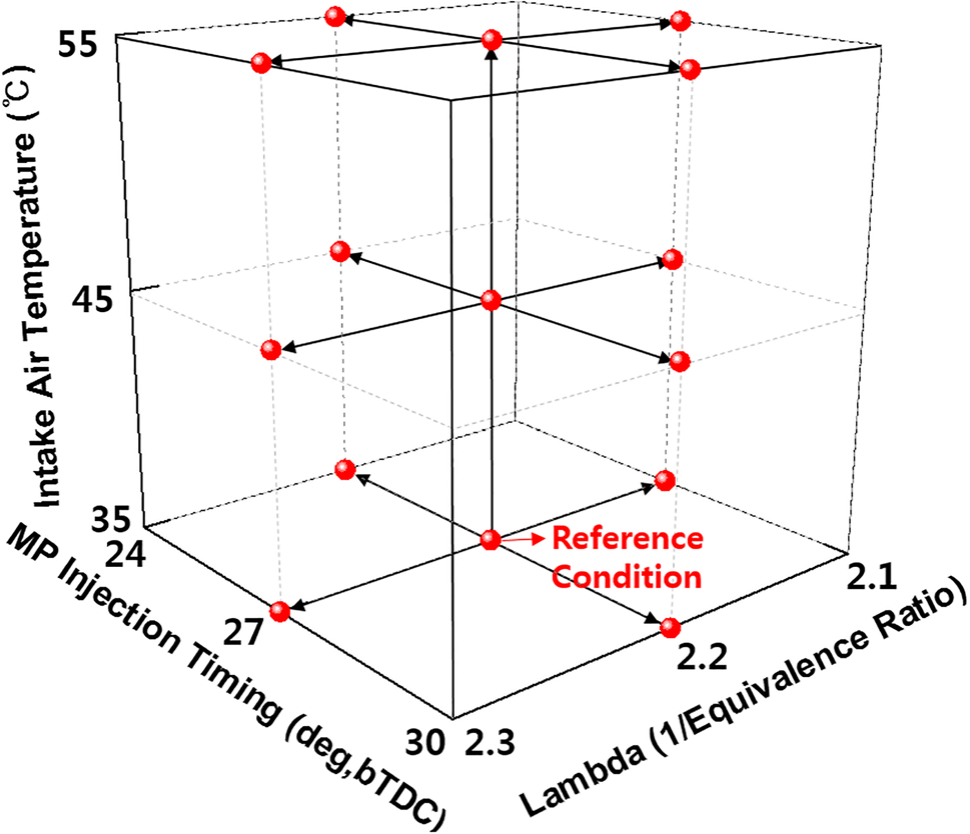
Experiment conditions for micro pilot (MP) injection timing, equivalence ratio, and intake air temperature.

In addition to engine experiments for the reference condition, additional experiments were conducted to derive the optimal engine operating conditions and premixed gas compositions. The MP injection timings, equivalence ratios, and intake air temperatures were varied for all premixed gas compositions. Figure 2 shows the conditions for the additional experiments. From the reference condition, the MP injection timings were varied in a range of ± 3° from the bTDC of 27°. Moreover, the lambda and intake air temperature were increased from the reference condition to ± 0.1 and + 20 °C in intervals of 10 °C, respectively. Therefore, 14 experimental data points were added for a given premixed gas composition.

### Quantification of the combustion intensity

In this study, the expected MPDF combustion forms were misfiring, premixed mixture ignition in the end-gas region (PREMIER) combustion, and knocking combustion, depending on the engine operating conditions and premixed gas compositions. In the existing studies, the ringing intensity (RI) was used to quantify the knocking combustion intensity^40–42^. Therefore, in this study, the RI was used as a quantitative indicator of the combustion intensity, and the characteristics of MPDF combustion were expressed in terms of RI, defined as follows:

4 $$Ringing\; Intensity \left( {MW/m^{2} } \right) = \frac{{\sqrt {\gamma RT_{max} } }}{{2\gamma P_{max} }}\left[ {\beta \left( {\frac{dP}{{dt}}} \right)_{max} } \right]^{2}$$

where $\sqrt {\gamma RT_{max} }$ is the speed of sound, and $dP/dt$ is the pressure gradient in the combustion process. Moreover, $\beta$ is a tuning parameter for correlating the pressure vibration, set as 0.05, and $\gamma$ is the specific heat ratio^40^. The following assumptions were considered to determine the RI: (1) When the intake valve opens, the average temperature of the combustion chamber is the same as the intake air temperature. (2) When the intake and exhaust valves close, mass does not move in or out of the combustion chamber. (3) The gas in the combustion chamber is an ideal gas. The acquired engine data were processed using the LabVIEW program, and the average temperature of the cylinder and RI were calculated.

## Results and Discussion

### Characteristics of MPDF combustion

**Figure 3:**
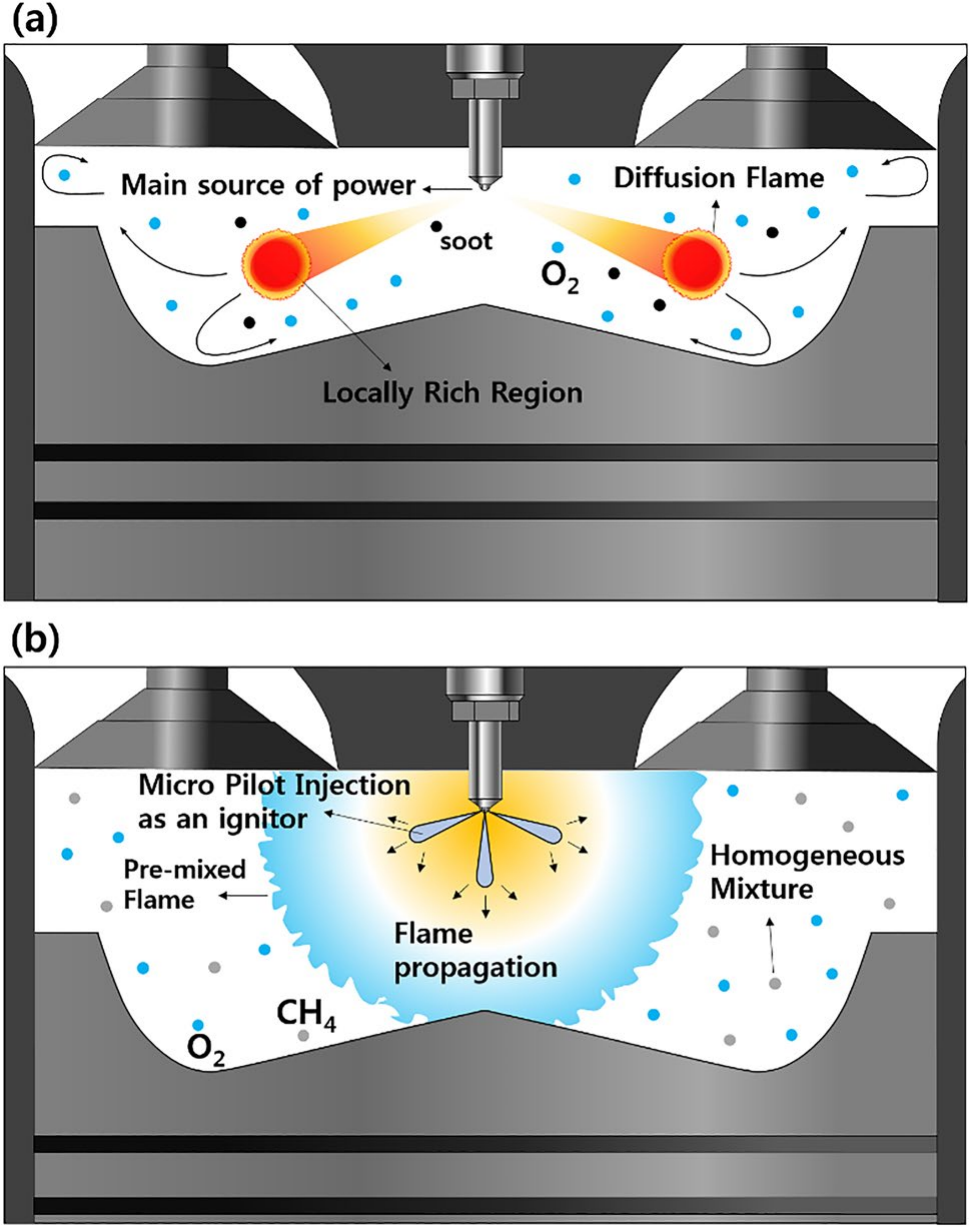
Comparison of combustion technologies: (**a**) diesel combustion and (**b**) MPDF combustion in a compressed ignition engine.

Before investigating the effects of the premixed gas composition on the MPDF combustion characteristics, the differences between diesel combustion and MPDF combustion in a CI engine must be clarified. Therefore, preliminary experiments were conducted with different CH_4_ and diesel mixture ratios in a CI engine. Figure 3a and b illustrate the process of diesel combustion and MPDF combustion in the CI engine. In diesel combustion, diesel is used as the main power source. Under diesel single-injection conditions, a low cylinder temperature increases the ignition delay and renders the mixture homogeneous, leading to diesel premixed combustion. If a large amount of diesel is ignited at once, diesel knocking combustion may occur. In diesel combustion, the pilot injection strategy is widely used to prevent knocking combustion and control the combustion reaction rate. After diesel premixed combustion in the initial phase, diffusion combustion predominantly occurs in the main combustion phase. In this period, NOx is formed in a high-temperature region in which the equivalence ratio approaches the stoichiometric condition. In contrast, soot is produced from a local high-mixture-ratio region. Unlike the diesel combustion mode, an extremely small amount of diesel fuel is used as an ignitor for MPDF combustion. Most of the energy is sourced from the premixed gas fuel. The injected diesel ignites the surrounding homogeneous mixture, forming premixed flame surfaces. Consequently, premixed combustion predominantly occurs in the MPDF condition. If the injected diesel does not promptly ignite and form a homogeneous mixture, the diesel does not function as the ignition source and leads to misfiring. Therefore, a high cylinder temperature must be maintained at the MP injection timing. However, auto-ignition may occur in the regions of high local temperature before the MP injection timing, resulting in knocking combustion. In this study, the optimum MP injection timing (bTDC 27°) and intake air temperature (35 °C) were derived by performing experiments for pure CH_4_ gas. In the MPDF combustion, both the NOx and PM emissions are significantly decreased because of the low combustion temperature and homogeneous mixture. However, the low combustion temperature leads to a large amount of incomplete combustion products, and HC and CO emissions are dramatically increased. This phenomenon also deteriorates the combustion efficiency.

**Figure 4:**
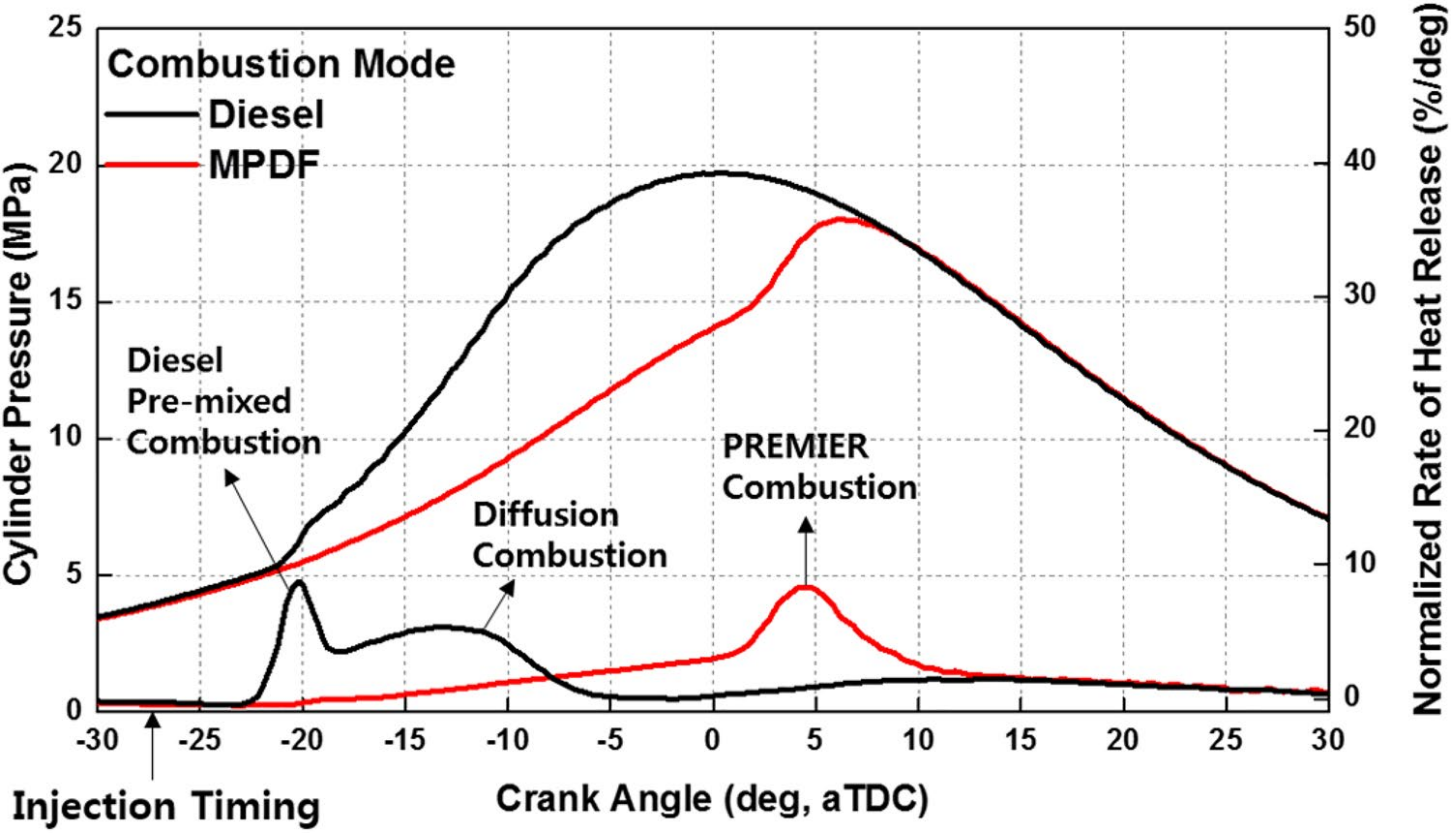
Cylinder pressure and normalized rate of heat release for diesel and MPDF combustion.

The experimental conditions for MPDF combustion are the same as the reference conditions, with an extremely small amount of diesel injected. In contrast, only diesel fuel was used in the diesel combustion mode, and the LHV of diesel was set as 3022 J/stroke, same as the LHV of the MPDF combustion. Figure 4 shows the cylinder pressure and normalized rate of heat release for the diesel combustion and MPDF combustion. To analyze the differences between the two types of combustion, the diesel injection timing was set a bTDC 27° in all experimental conditions. Because the fuel injection timing is optimized for MPDF combustion, the position of the peak cylinder pressure is not reasonable, and the heat release rate is concentrated in the compression stroke in the pure diesel combustion mode. The highly advanced combustion phasing can be attributed to the high reactivity of diesel, and the fuel injection timing approaches the TDC in the diesel combustion mode. In the single diesel injection condition, increased ignition delay leads to a large amount of premixed mixture, inducing high-intensity premixed combustion. The subsequent combustion mode is characterized by a lower combustion intensity and higher combustion duration than that of premixed combustion. In comparison, the MPDF combustion condition exhibits an increased ignition delay and lower combustion intensity because of the small amount of injected diesel. Therefore, high-intensity premixed combustion occurs in the middle of the combustion.

**Figure 5:**
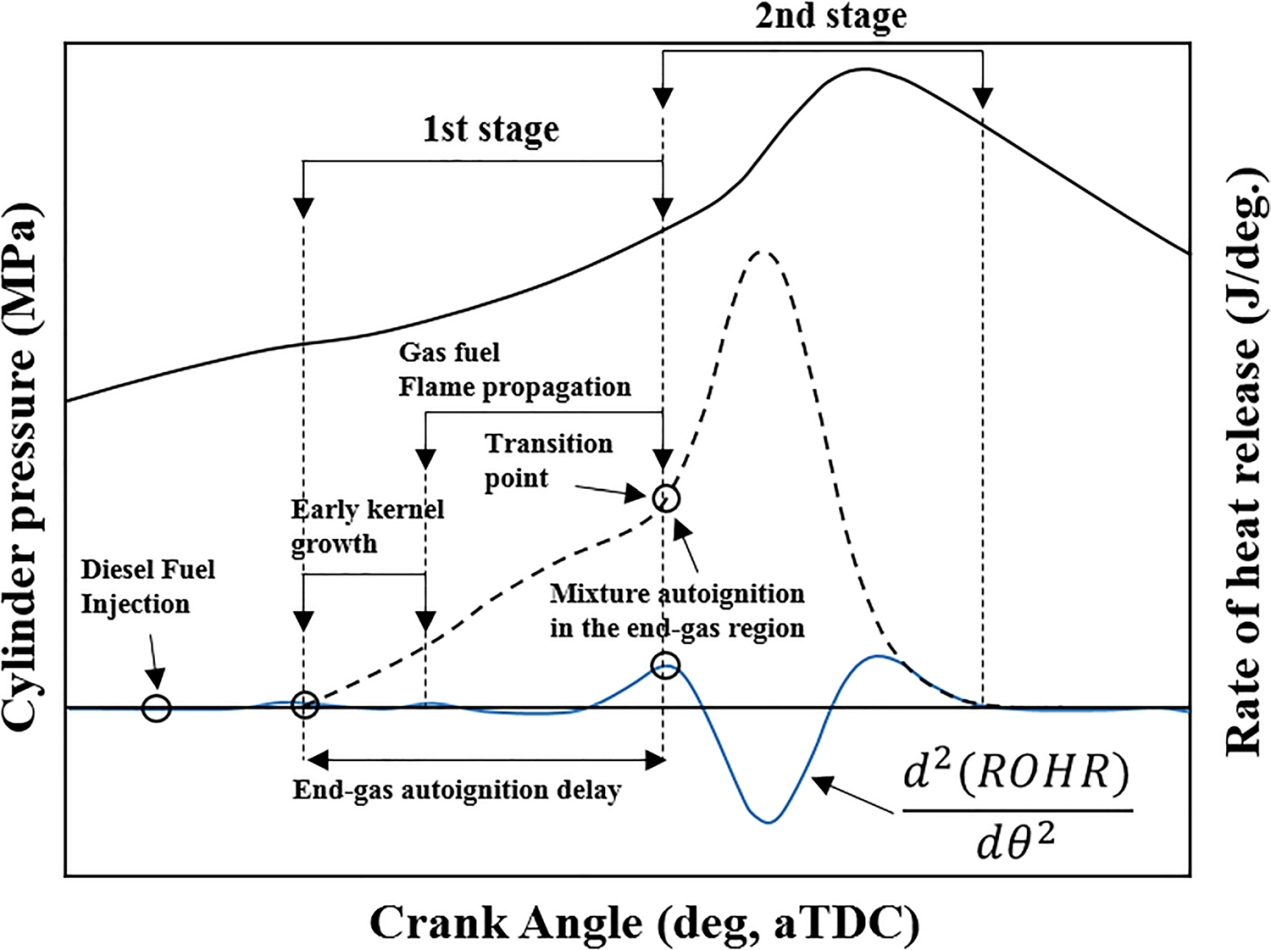
Concept of PREMIER combustion^43^.

In this study, the MPDF combustion is dominated by PREMIER combustion, as shown in Fig. 5^43^. PREMIER combustion is more stable than incomplete and knocking combustion^27,44,45^. According to Azimov et al.^44^, the characteristics of end-gas mixture auto ignition under PREMIER combustion differ from those of knocking combustion. In the existing studies, to analyze MPDF combustion, the second derivative of rate of heat release was used to distinguish the transition from the first combustion stage to the second combustion stage ^43,44,46,47^. In contrast, in this study, the combustion stages were divided considering the MFB, based on the CA. The combustion periods were defined from MFB CA10, corresponding to the end of the ignition delay, to CA90, corresponding to the end of combustion. In addition, the combustion periods were divided into the main and residual gas combustion periods, corresponding to MFB CA10–70 and MFB CA70–90, respectively.

**Figure 6:**
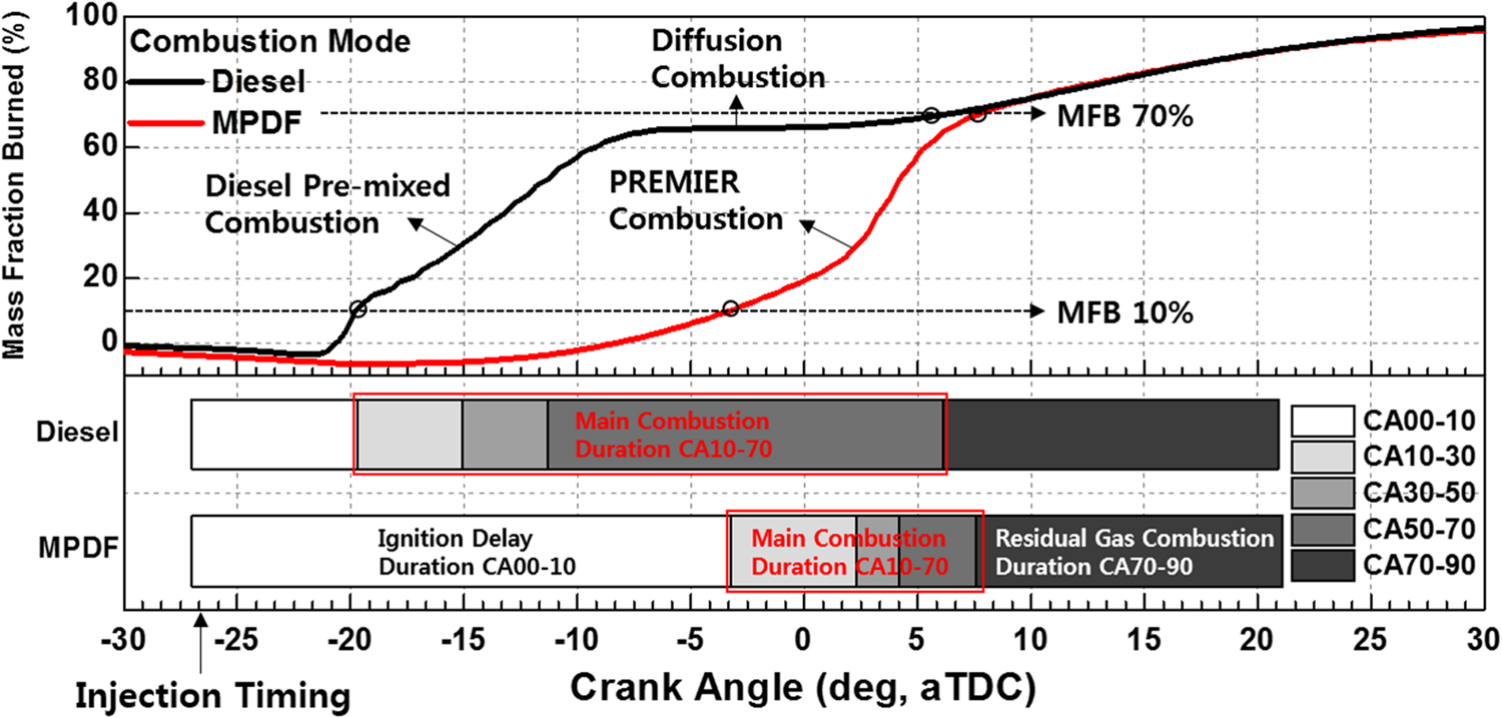
MFB for diesel and MPDF combustion.

Figure 6 clearly shows the difference in the combustion rates of diesel combustion and MPDF combustion. The MFB CA10 of diesel combustion and MPDF combustion is considerably different. In the diesel combustion mode, the presence of highly reactive diesel promotes the combustion, with an ignition delay of CA 8°. Because an extremely small amount of diesel is used in MPDF combustion, the ignition delay increases to CA 24°. The trends of the main combustion period are opposite to those of the ignition delay. As mentioned, a lower intensity of diffusion combustion corresponds to an enlarged main combustion period. In MPDF combustion, a low combustion rate is observed at the beginning of combustion (MFB CA10). However, the combustion rate significantly increases after MFB CA20, when auto-ignition occurs in the end gas region, and this rate persists until MFB CA70. Therefore, PREMIER combustion effectively decreases the main combustion periods compared to those in diesel combustion. After MFB CA70, the diesel combustion and MPDF combustion exhibit similar residual gas combustion rates.

**Figure 7:**
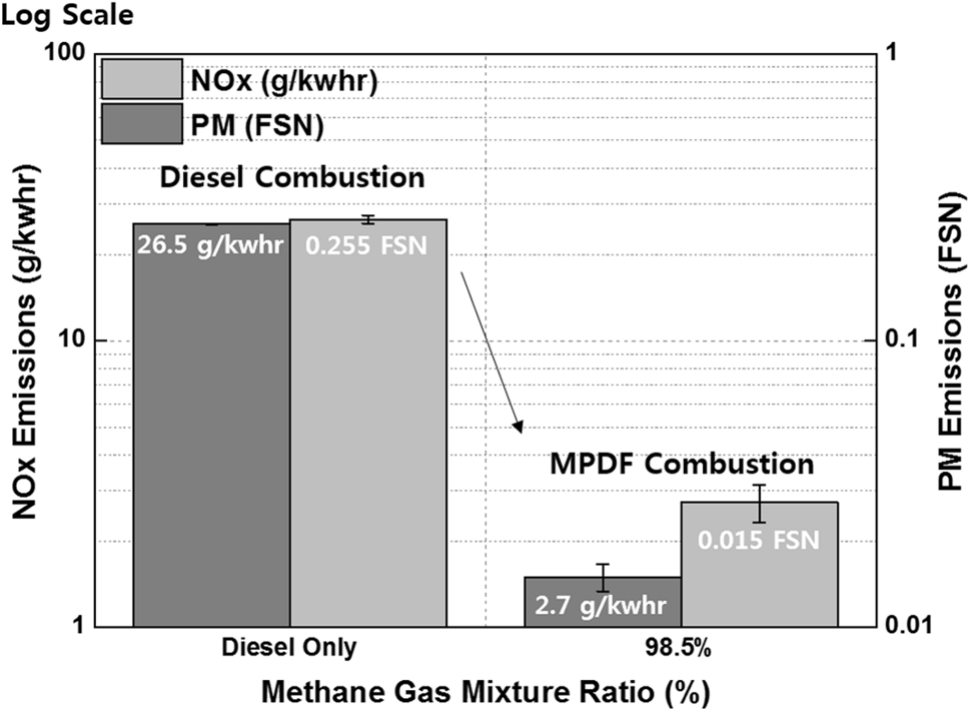
NOx and PM emissions for diesel and MPDF combustion.

Figure 7 illustrates the explanations for the differences in diesel combustion and MPDF combustion. The amount of NOx and PM emissions in diesel combustion is considerably larger than that in MPDF combustion. Considering these advantages of MPDF combustion, several researchers have focused on the use of gaseous fuel as an alternative to diesel. In this study, the effects of premixed gas compositions on the MPDF combustion was examined, as described in the subsequent section, to prevent misfiring and knocking combustion.

### Effects of the C_3_H_8_ mixture ratio on MPDF combustion

**Figure 8:**
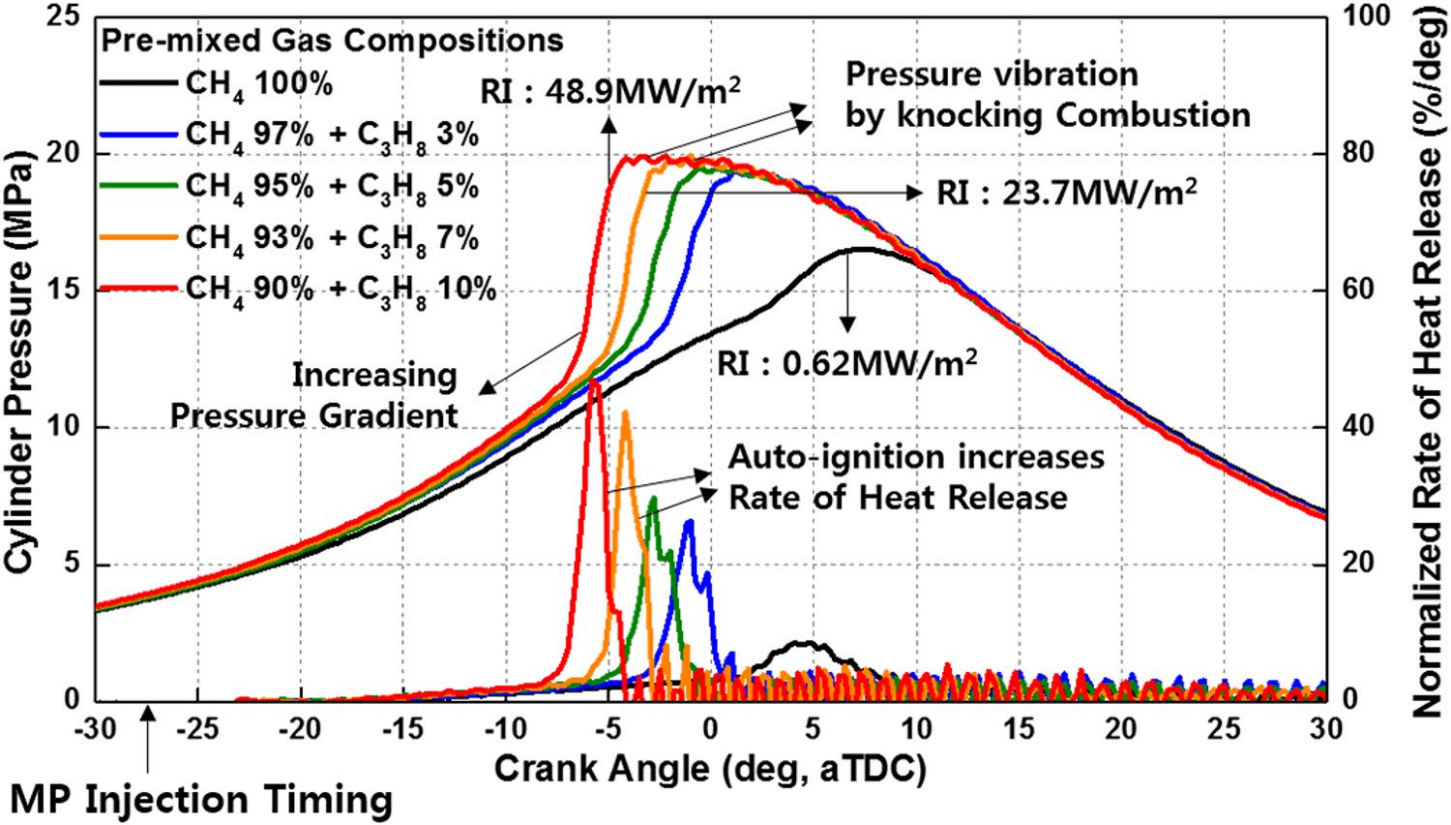
Variation in the cylinder pressure and normalized rate of heat release with the C_3_H_8_ mixture ratio.

**Figure 9:**
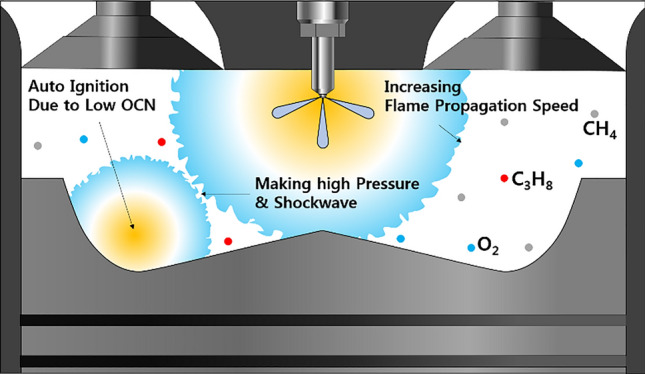
Schematic of effects of C_3_H_8_ on MPDF combustion.

To analyze the effects of C_3_H_8_ on the MPDF combustion characteristics, the C_3_H_8_ mixture ratio was varied, and the other experimental conditions were set as the reference condition. Figure 8 shows the variation in the cylinder pressure and normalized rate of heat release with the C_3_H_8_ mixture ratio. As the C_3_H_8_ mixture ratio increases, the combustion phasing is advanced, and the combustion intensity increases. When the C_3_H_8_ mixture ratio exceeds 7%, the peak normalized rate of heat release is significantly increased owing to auto-ignition in the local high-temperature mixture regions. Therefore, knocking combustion occurs with fluctuations in the cylinder pressure. According to an existing study on hydrocarbon gas characteristics, with the increasing numbers of carbon components (from CH_4_ to C_3_H_8_ and C_4_H_10_), the methane number and OCN decrease^39^. In high-temperature conditions, C_3_H_8_ and C_4_H_10_ can easily auto-ignite owing to their low OCNs and cause knocking combustion. Figure 9 illustrates the phenomenon of knocking combustion caused by C_3_H_8_. At increased mixture ratios of C_3_H_8_ in the premixed gas, auto-ignition occurs in local high-temperature regions. As the developing flame surfaces, the flame surfaces collide, and high pressure and shockwaves are generated, leading to considerable noise and engine vibrations. Similar to knocking combustion, PREMIER combustion generates a high-pressure gradient from the auto-ignition of the end gas. However, the noise and vibration associated with PREMIER combustion are less notable than those of knocking combustion. The RI was calculated to distinguish PREMIER and knocking combustion modes.

**Figure 10:**
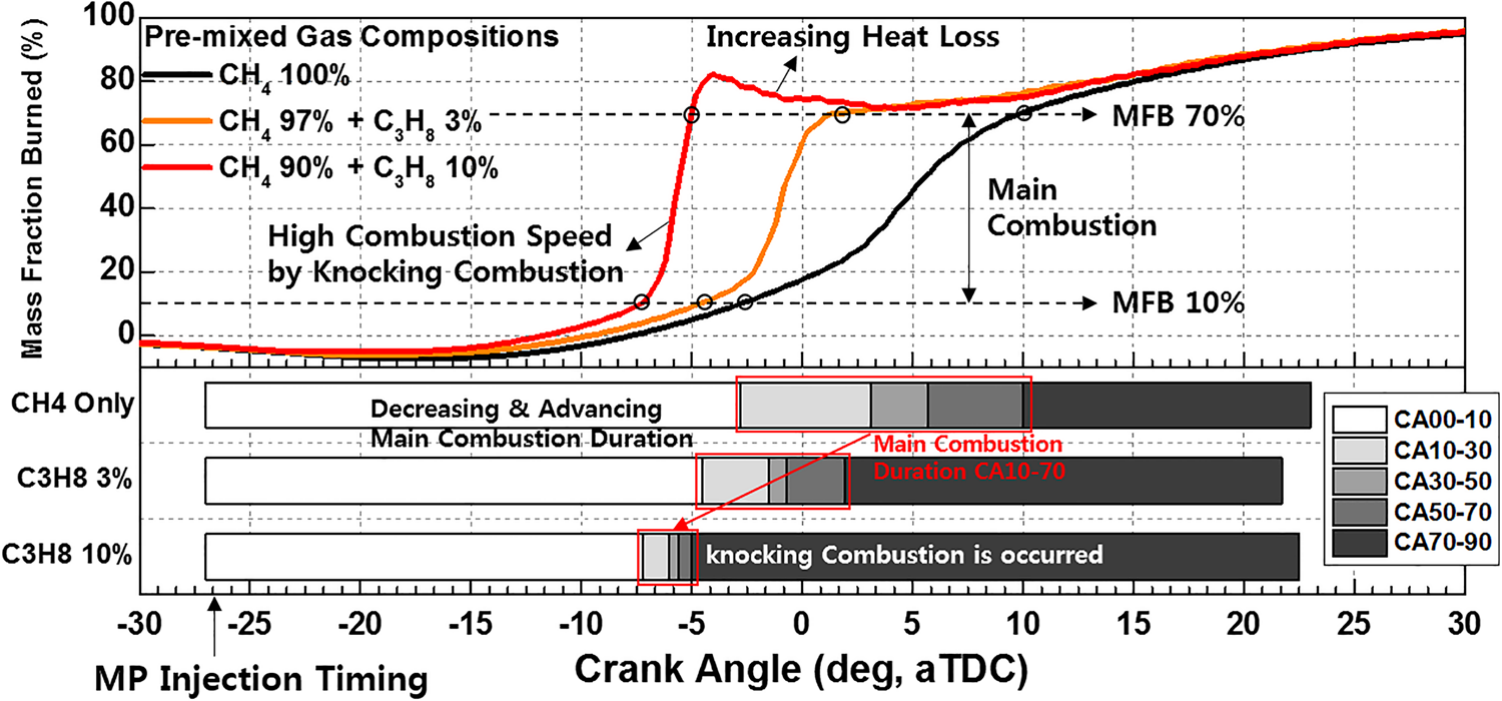
Variation in the MFB with the C_3_H_8_ mixture ratio.

Figure 10 shows the variation in the MFB with the C_3_H_8_ mixture ratio in MPDF combustion. In addition to the ignition delay, the main combustion duration (MFB CA10–70) decreases and is advanced at higher C_3_H_8_ mixture ratios. In particular, when the C_3_H_8_ mixture ratio is 10% and knocking combustion occurs, the main combustion duration is significantly lower than that at other ratios. Moreover, because of knocking combustion, the cylinder temperature increases, leading to an increase in various heat losses (including heat transfer loss, coolant loss, and exhaust loss), resulting in lower accumulated heat release. Moreover, the effects of the C_3_H_8_ mixture ratio on the MPDF combustion can also be clarified considering the engine performances.

**Figure 11:**
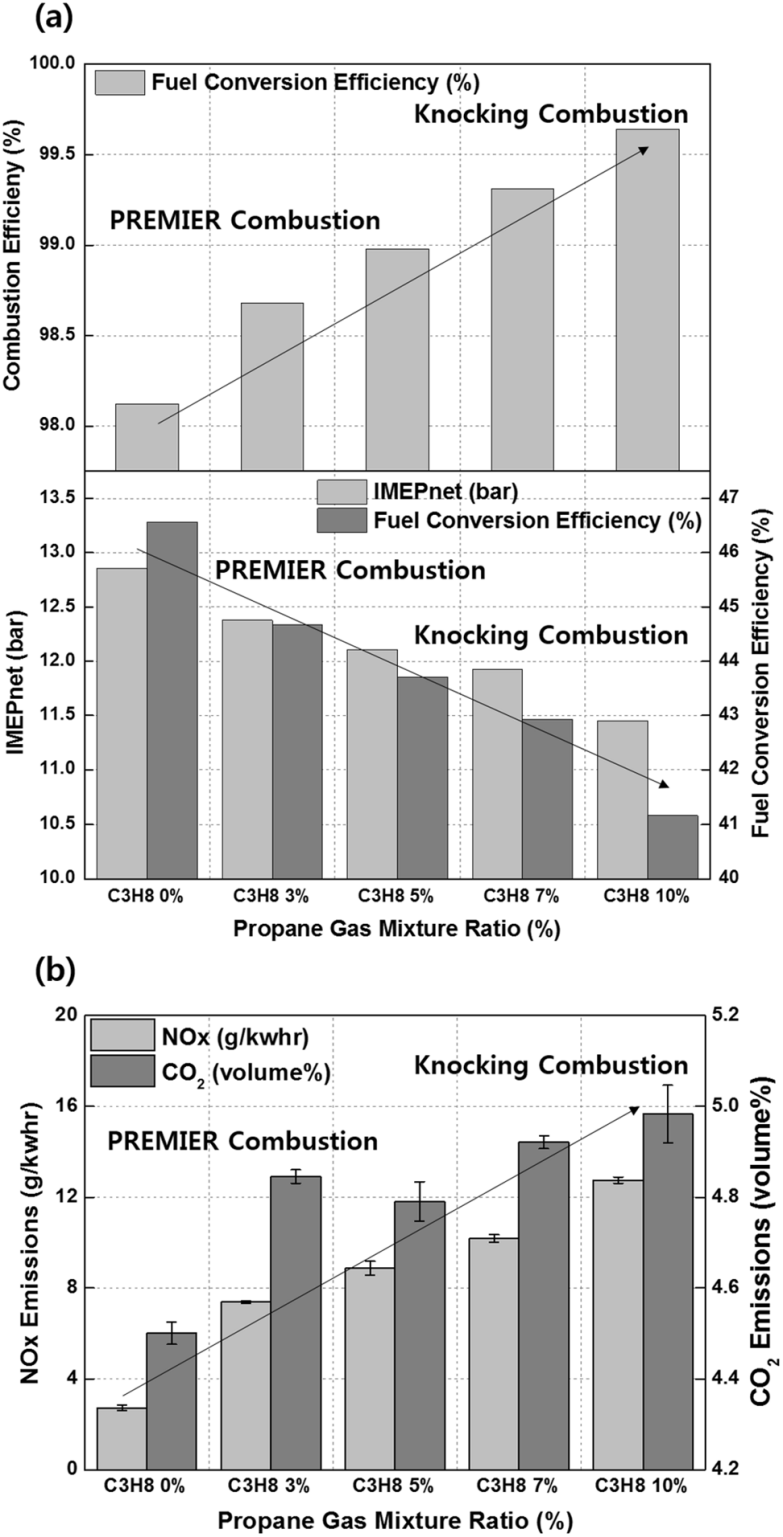
Effects of the C_3_H_8_ mixture ratio on the (**a**) combustion efficiency, IMEPnet, and fuel conversion efficiency and (**b**) NOx and CO_2_ emissions.

**Figure 12:**
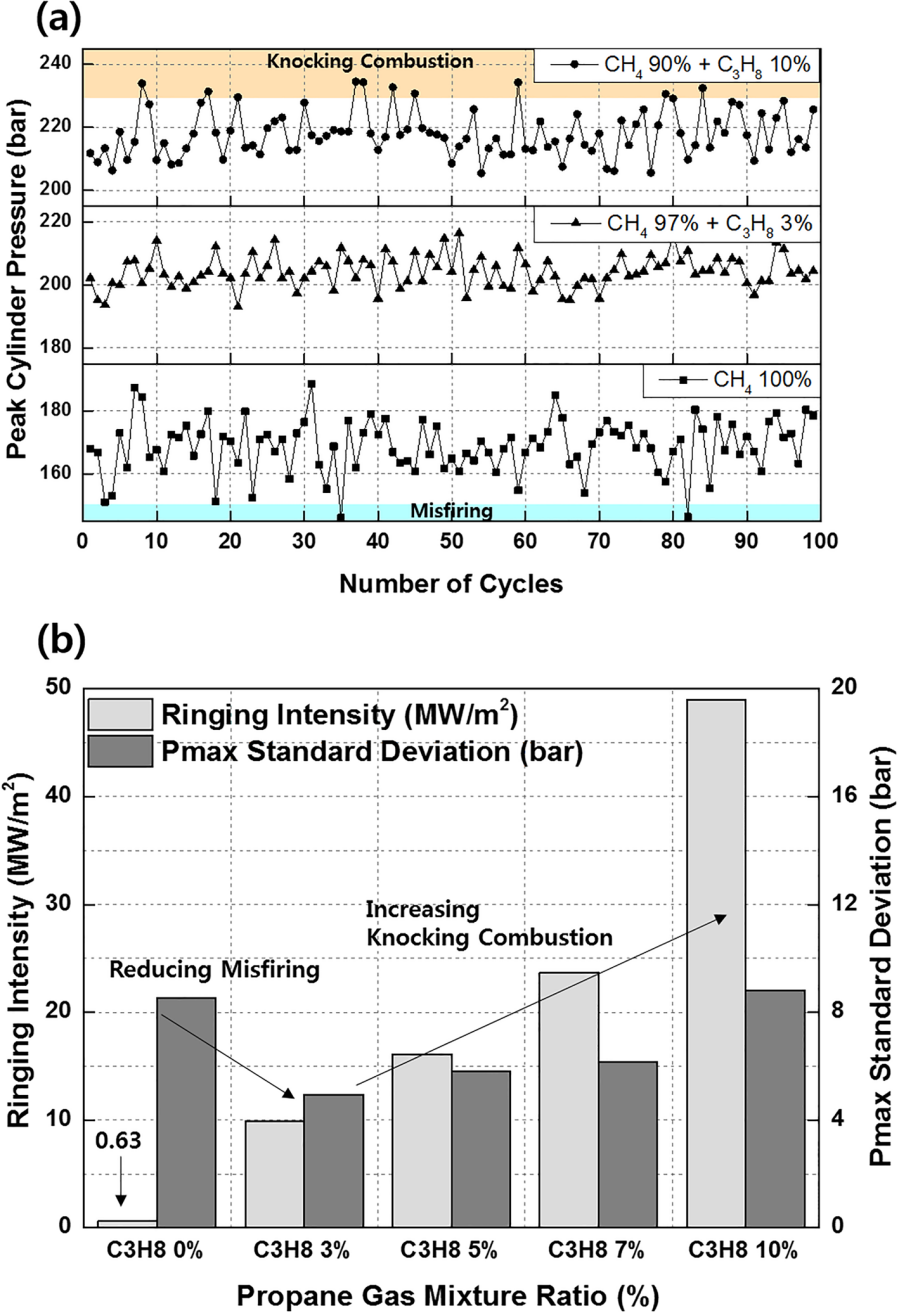
Effects of the C_3_H_8_ mixture ratio on the (**a**) cycle to cycle variation in the peak cylinder pressure and (**b**) RI and STD of the peak cylinder pressure.

Figure 11a shows the effects of the C_3_H_8_ mixture ratio on the combustion efficiency, net indicated mean effective pressure (IMEPnet), and fuel conversion efficiency. Increasing the C_3_H_8_ mixture ratio helps prevents incomplete combustion by promoting MPDF combustion. However, increasing the C_3_H_8_ mixture ratio impairs the engine performance by advancing the combustion phasing. Even when the C_3_H_8_ mixture ratio is 10%, the main combustion is completed before TDC, generating negative work. In addition to the various heat losses, the increasing negative work deteriorates the IMEPnet and fuel conversion efficiency. Figure 11b shows that the presence of C_3_H_8_ promotes MPDF combustion. Because MPDF combustion generates a large amount of incomplete combustion produces owing to the low combustion temperature, the detection limit of the exhaust gas analyzer is exceeded. In this case, the incomplete combustion rate can be indirectly analyzed by measuring the CO_2_ emissions. The NOx and CO_2_ emissions steadily increase with the addition of C_3_H_8_. As mentioned, C_3_H_8_ effectively promotes MPDF combustion, and the increasing combustion temperature leads to high NOx and CO_2_ emissions. Furthermore, as the C_3_H_8_ mixture ratio increases, the carbon number in the premixed gas increases under the same experimental conditions, leading to increased CO_2_ emissions. Many researchers have used the cycle-to-cycle variation in the peak cylinder pressure to determine the combustion stability. In this study, the variations in the peak cylinder pressure were determined to analyze the effects of premixed gas compositions on the combustion stability. Specifically, 100 cycles of data were measured and averaged for one experimental case, and the peak cylinder pressure was calculated as one value per cycle. Figure 12a shows the 100 cycles of peak cylinder pressure and its variation with the C_3_H_8_ mixture ratio. The combustion variation is intensified by misfiring and knocking combustion. Because pure CH_4_, which has a high OCN, has a higher auto-ignition point than C_3_H_8_, unpredictable auto-ignition is not observed in the pure CH_4_ condition, although it occurs in the cases of the CH_4_–C_3_H_8_ premixed gases. Nevertheless, misfiring, in which the flame surface does not develop normally from the ignition point, occurs because of the lower flame propagation speed of CH_4_ than that of C_3_H_8_. When the C_3_H_8_ mixture ratio is 10%, the low auto-ignition temperature causes knocking combustion, resulting in a considerably higher peak cylinder pressure. Figure 12b shows the variations in the RI and standard deviation (STD) of the peak cylinder pressure with the C_3_H_8_ mixture ratio. The RI of pure CH_4_ is the lowest and corresponds to the largest combustion variation because of misfiring. The misfiring phenomenon disappears as the C_3_H_8_ mixture ratio increases, and thus, the STD of the peak cylinder pressure, which represents the combustion variation, decreases. Therefore, the lowest combustion variation is observed, without any misfiring and knocking combustion, when the C_3_H_8_ mixture ratio is 3%. As the C_3_H_8_ mixture ratio increases, the combustion form changes from PREMIER combustion to knocking combustion, and the RI and STD of the peak cylinder pressure increase. These results demonstrate that the addition of a small amount of C_3_H_8_ can help enhance the combustion stability under low-load conditions in which misfiring occurs.

### Effects of the H_2_ mixture ratio on MPDF combustion

**Figure 13:**
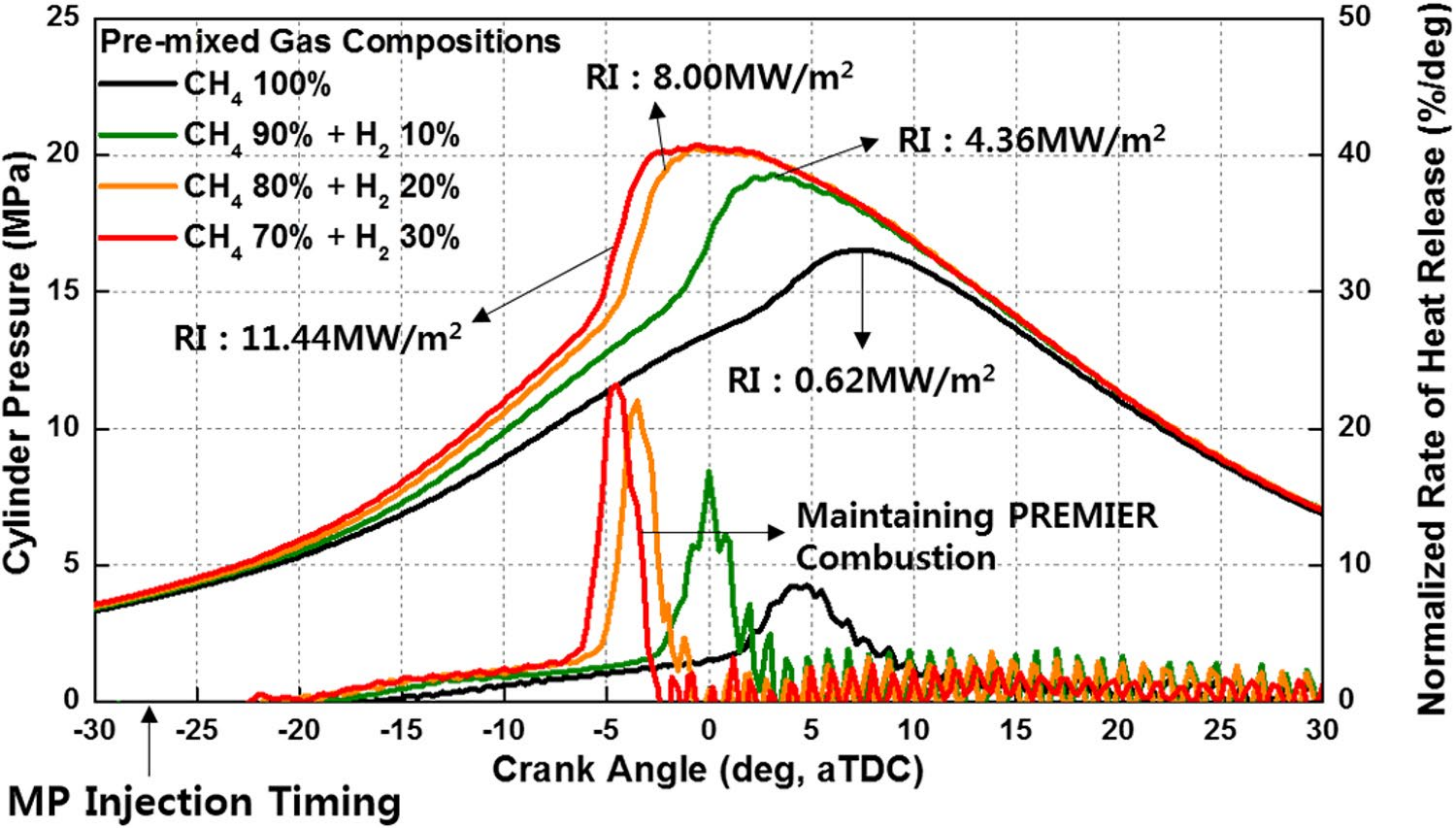
Variation in the cylinder pressure and normalized rate of heat release with the H_2_ mixture ratio.

Engine experiments were performed to analyze the effects of H_2_ on the MPDF combustion characteristics. The H_2_ mixture ratio was varied from 0 to 30% in intervals of 10%. Figure 13 shows the variation in the cylinder pressure and normalized rate of heat release with the H_2_ mixture ratio. C_3_H_8_ and H_2_ tend to promote MPDF combustion. Because the flame propagation speed of H_2_ is higher than that of CH_4_, advancement of the combustion phasing and increased combustion intensity are observed in conditions with higher H_2_ mixture ratios^33^. However, the effects of C_3_H_8_ and H_2_ are considerably different in terms of knocking combustion. Even at high H_2_ mixture ratios, a high pressure gradient and fluctuation of the cylinder pressure caused by knocking combustion are not observed, and the PREMIER combustion form is maintained.

**Figure 14:**
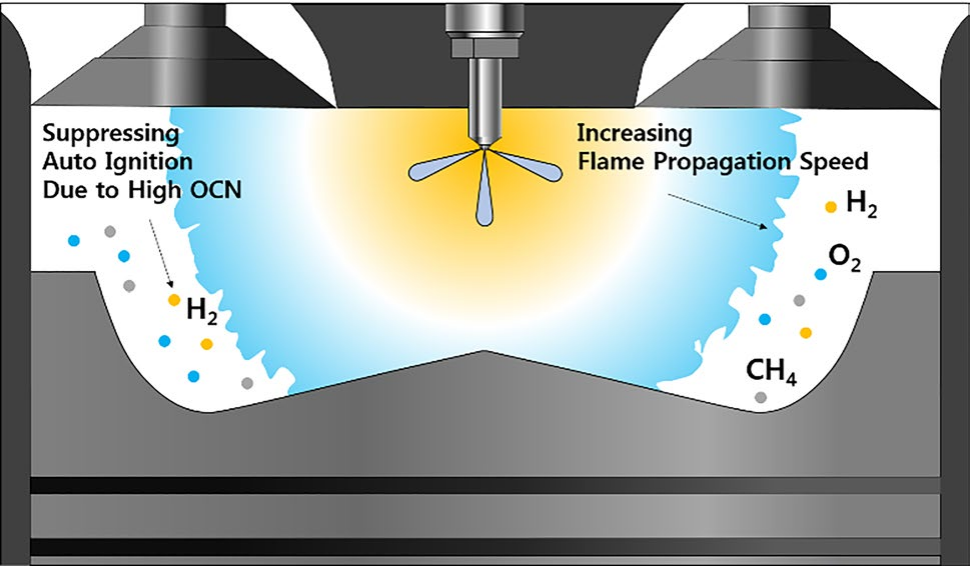
Schematic of the effects of H_2_ on MPDF combustion.

Figure 14 schematically illustrates the effects of H_2_ on MPDF combustion. Unlike C_3_H_8_, H_2_ has a high OCN, which helps prevent auto-ignition, which is a source of knocking combustion^34^. The high flame propagation speed of H_2_ decreases the ignition delay and combustion period, and the engine noise and vibration caused by knocking combustion can be alleviate by increasing the H_2_ mixture ratio in the premixed gas.

**Figure 15:**
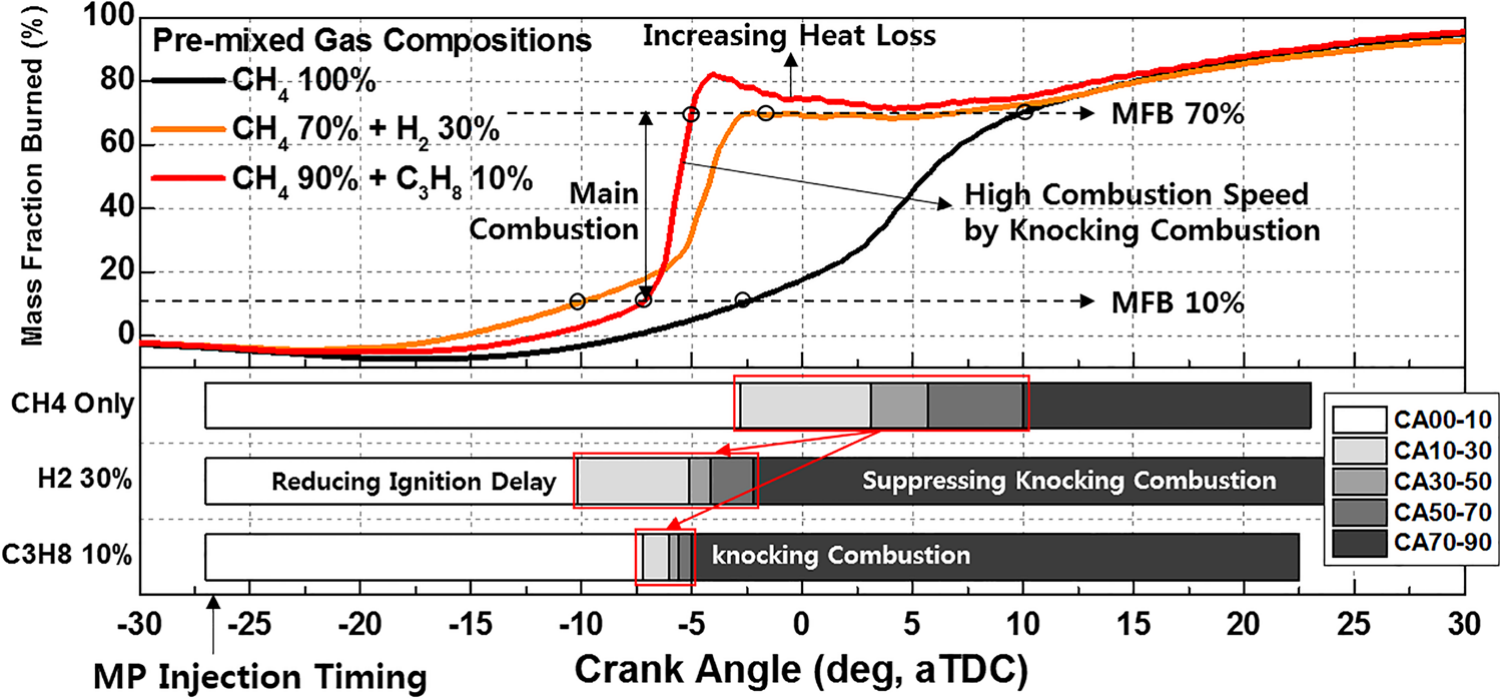
Variation in the MFB with the C_3_H_8_ and H_2_ gas mixture ratios.

Figures 15 provides an explanation for the differences in the effects of C_3_H_8_ and H_2_ on MPDF combustion. The ignition delay decreases with the increase in the H_2_ mixture ratio, and the highest combustion rate until MFB CA 20 is observed when the H_2_ mixture ratio is 30%, because H_2_ accelerates the flame surfaces propagation. However, a significantly high combustion intensity is observed after MFB CA 20–70 when the C_3_H_8_ mixture ratio is 10%. C_3_H_8_ leads to knocking combustion, which decreases the main combustion duration. In contrast, H_2_ moderately decreases the combustion rate by suppressing knocking combustion. Therefore, the main combustion duration when the H_2_ mixture ratio is 30% is larger than that when the C_3_H_8_ mixture ratio is 10%. Moreover, the differences at MFB CA 90 are minimal, which indicates that the end timing of combustion is not influenced by the premixed gas compositions. By replacing C_3_H_8_ with H_2_ to prevent knocking combustion, various heat losses can be decreased. The differences in the effects of H_2_ and C_3_H_8_ gases on the combustion characteristics are further highlighted by the following results.

**Figure 16:**
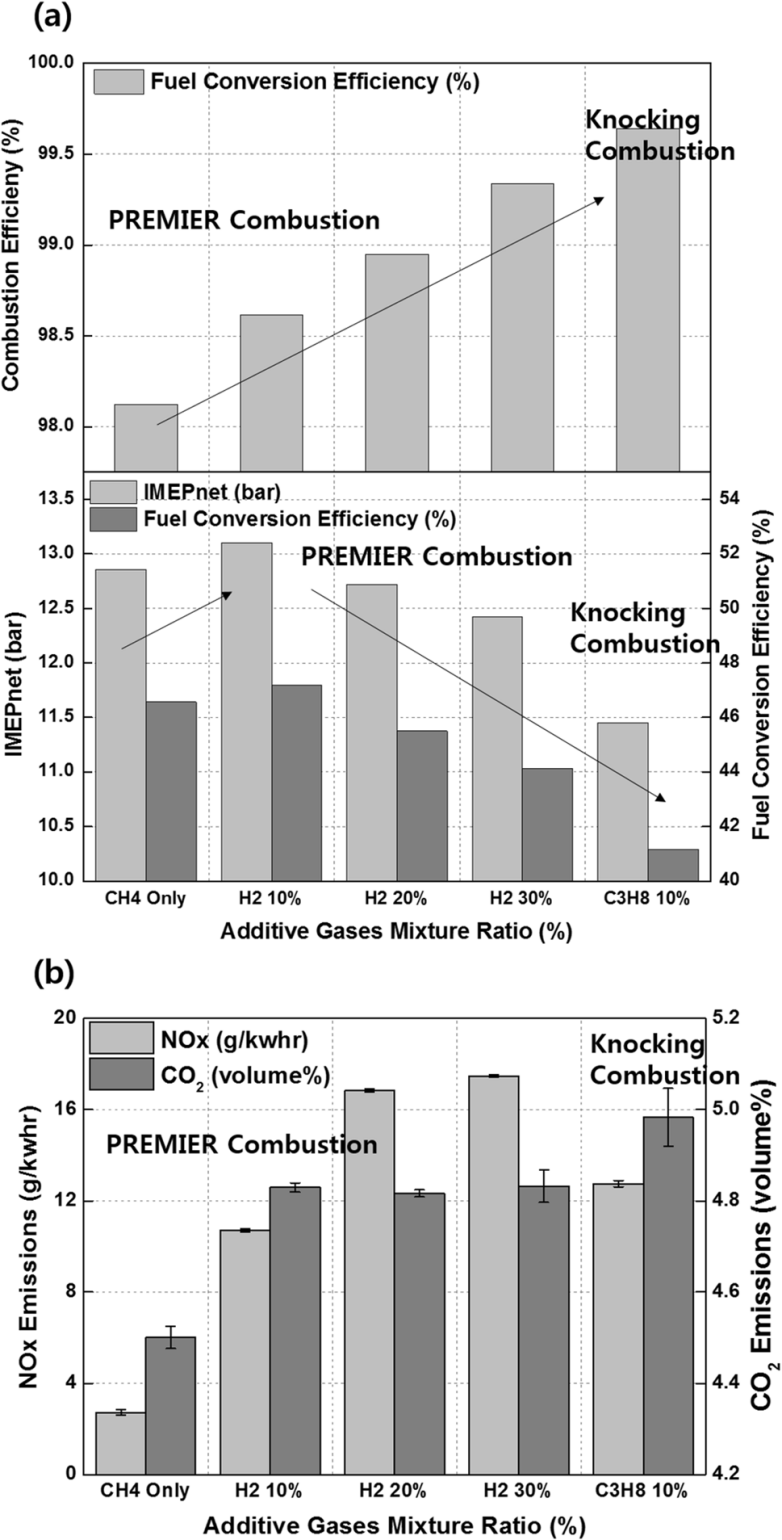
Effects of the C_3_H_8_ and H_2_ gas mixture ratios on the (**a**) combustion efficiency, IMEPnet, and fuel conversion efficiency and (**b**) NOx and CO_2_ emissions.

**Figure 17:**
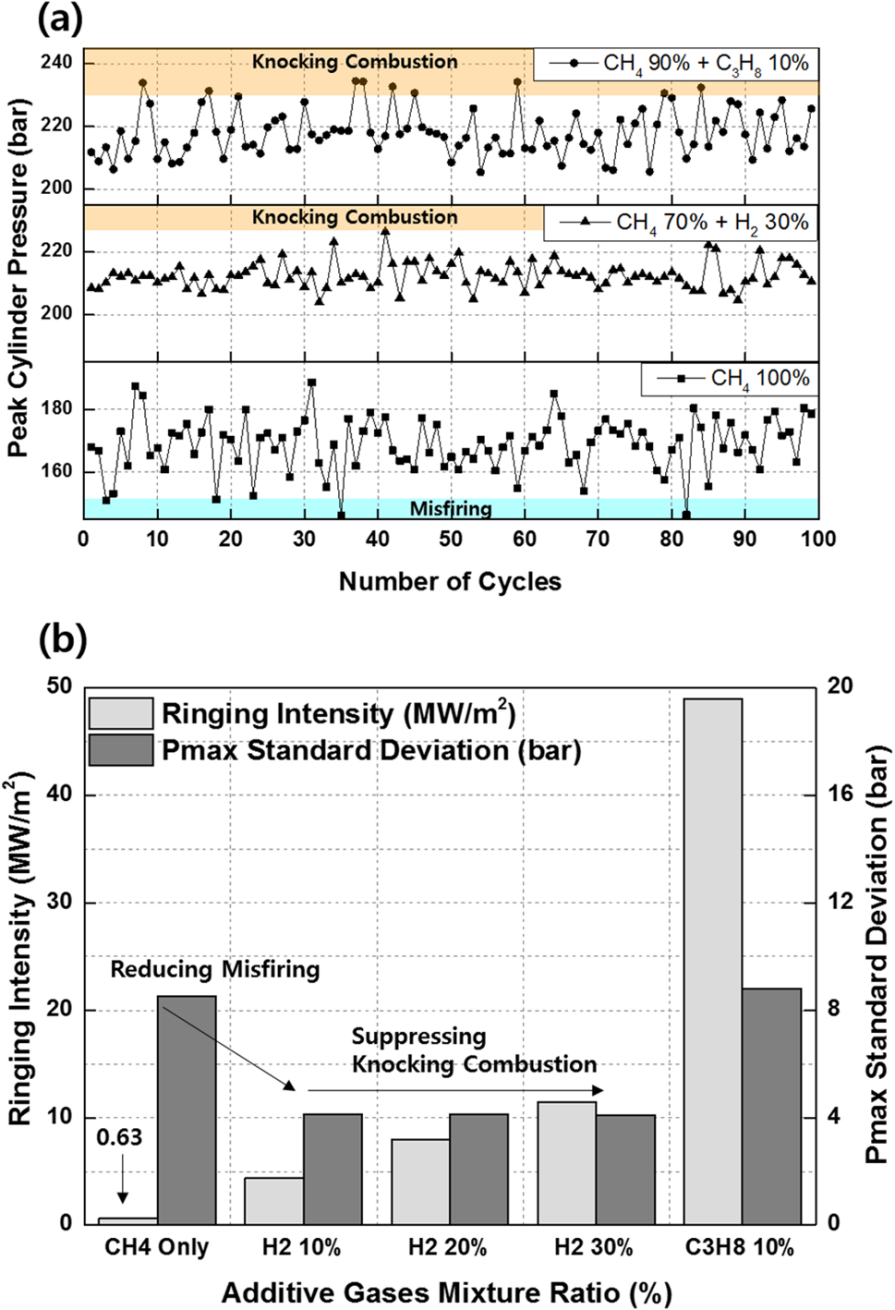
Effects of the C_3_H_8_ and H_2_ gas mixture ratios on the (**a**) cycle to cycle variation in the peak cylinder pressure and (**b**) RI and STD of the peak cylinder pressure.

Figure 16a shows the variations in the combustion efficiency, IMEPnet, and fuel conversion efficiency with the premixed gas compositions. Similar to C_3_H_8_, H_2_ promotes MPDF combustion and increases the combustion efficiency. The highest fuel conversion efficiency is observed when the H_2_ mixture ratio is 10%, attributable to the enhancement of incomplete combustion. With further increase in the H_2_ mixture ratio, the fuel conversion efficiency decreases, similar to the effect of C_3_H_8_ on the fuel conversion efficiency. The variations in the NOx and CO_2_ emissions with the premixed gas compositions are shown in Fig. 16b. The trends of the NOx emissions are consistent with the combustion phasing. The NOx emissions when the H_2_ mixture ratio is 30% are higher than those when the C_3_H_8_ mixture ratio is 10%. As shown in Fig. 15, the presence of H_2_ gas advances the combustion phasing and increases the temperature of the combustion chamber earlier. Therefore, the periods of NOx formation increase, resulting in high NOx emissions. The pure CH_4_ case corresponds to the lowest CO_2_ emissions because the low combustion temperature leads to a large amount of incomplete combustion products. H_2_ increases the CO_2_ concentration in the emission gases by promoting MPDF combustion. In addition to the combustion temperature, the carbon in the premixed gas affects the CO_2_ formation. Therefore, C_3_H_8_, which has three times the carbon than that in CH_4_ under the same volume, corresponds to the highest CO_2_ emissions. Figures 17a and b show that H_2_ enhances the combustion stability. Specifically, Fig. 17a illustrates the 100 cycles of peak cylinder pressure when pure CH_4_, H_2_ 30%, and C_3_H_8_ 10% are used. Misfiring and knocking combustion occur in the case of pure CH_4_ and C_3_H_8_ 10%, respectively. In contrast, when the H_2_ mixture ratio is 30%, PREMIER combustion is observed. Thus, the combustion variation can be considerably decreased by increasing the H_2_ mixture ratio. Figure 17b shows the variation in the RI and STD of the peak cylinder pressure with the premixed gas compositions. The lowest combustion variation is observed when the H_2_ mixture ratio is 10–30%. Even at higher H_2_ mixture ratios, the RI and combustion variation are low, because H_2_ suppresses knocking combustion. The results demonstrate that H_2_ can effectively enhance the combustion stability. However, owing to its low density, large amounts of H_2_ are required to enhance the combustion stability and achieve the same level of power as that provided by hydrocarbon gases.

### Effects of the CO_2_ mixture ratio on MPDF combustion

**Figure 18:**
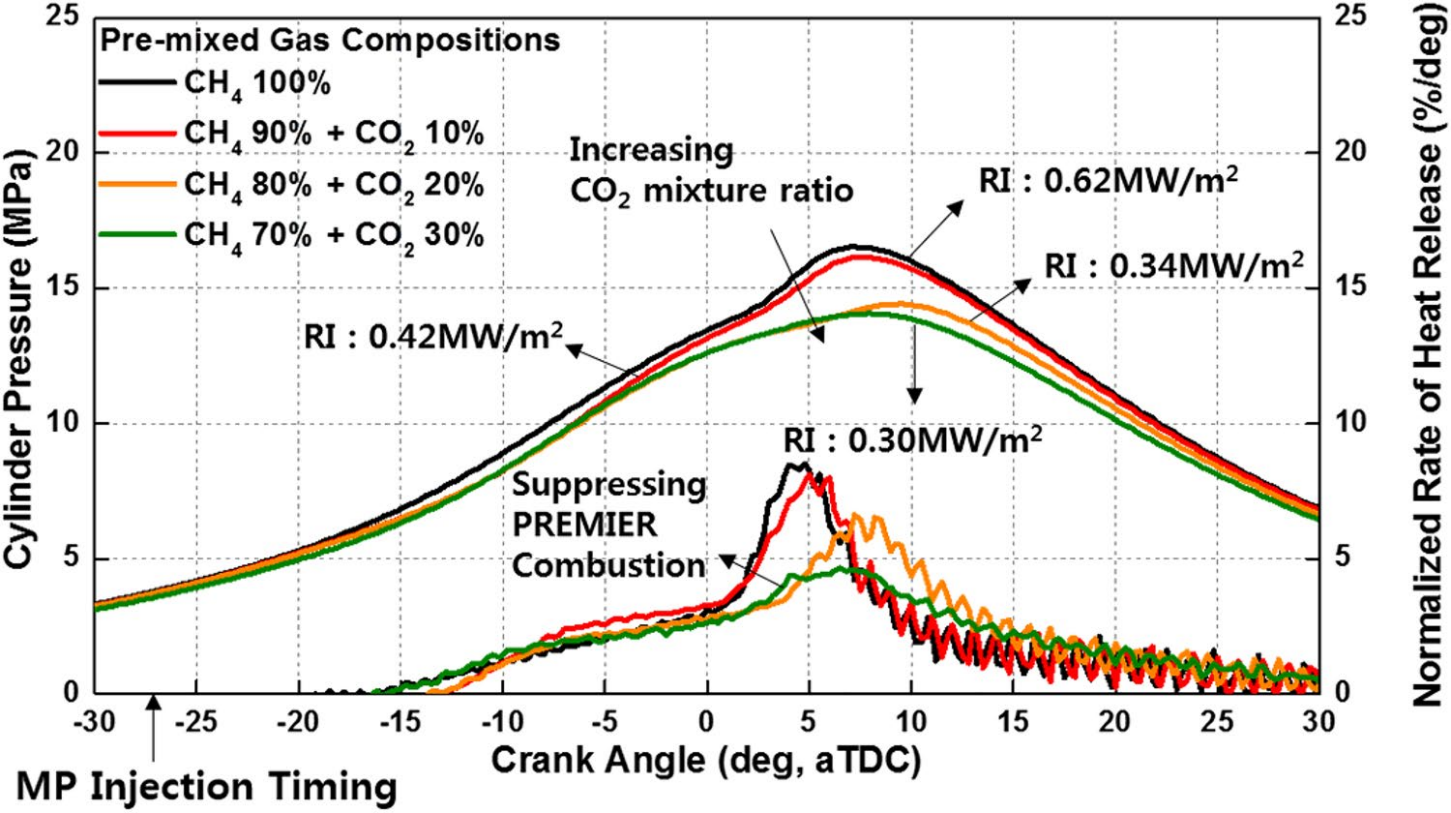
Variation in the cylinder pressure and normalized rate of heat release with the CO_2_ mixture ratio.

**Figure 19:**
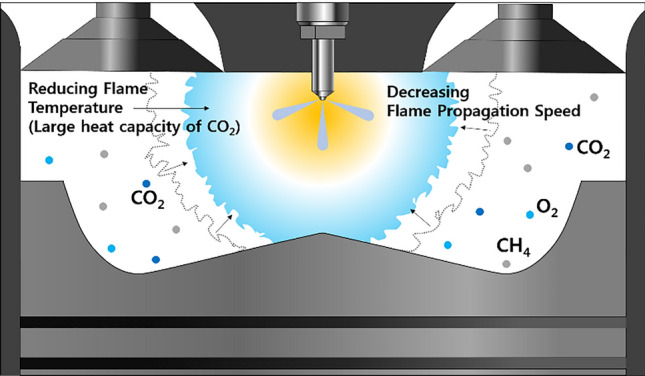
Schematic of the effects of CO_2_ on MPDF combustion.

This section describes the results of experiments in which the mixture ratio of CO_2_ was changed from 0 to 30% in intervals of 10%. Figure 18 shows the variation in the cylinder pressure and normalized rate of heat release with the CO_2_ mixture ratio. A higher CO_2_ mixture ratio corresponds to a lower combustion intensity and higher combustion duration. Notably, CO_2_ decreases the peak cylinder pressure and combustion intensity. Consequently, the PREMIER combustion is not observed when the CO_2_ mixture ratio is 30%. Unlike C_3_H_8_ and H_2_, CO_2_ suppresses MPDF combustion, as illustrated in Fig. 19. Owing to the large heat capacity of CO_2_, an increase in the mixture ratio of CO_2_ in the premixed gas decreases the combustion temperature, similar to the case in which the EGR rate is increased^37,38^.

**Figure 20:**
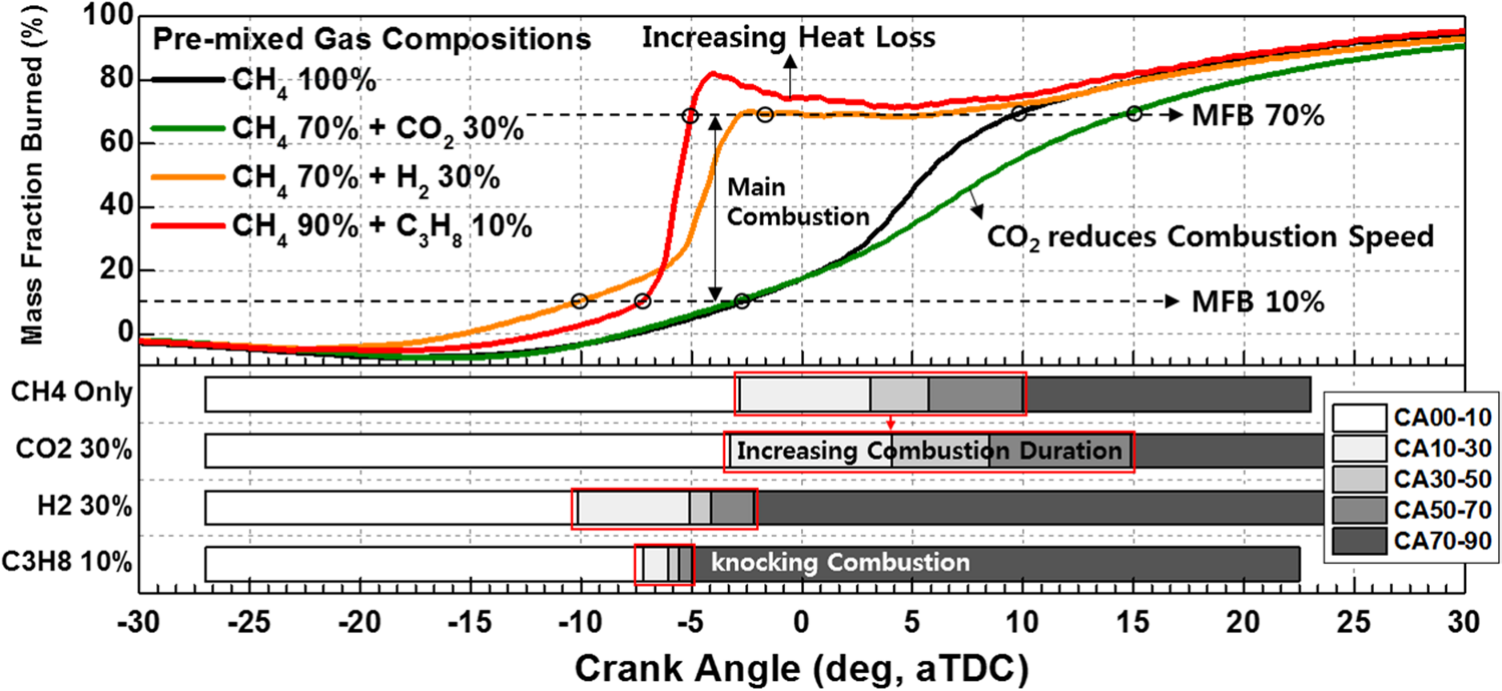
Variation in the MFB with the premixed gas compositions.

**Figure 21:**
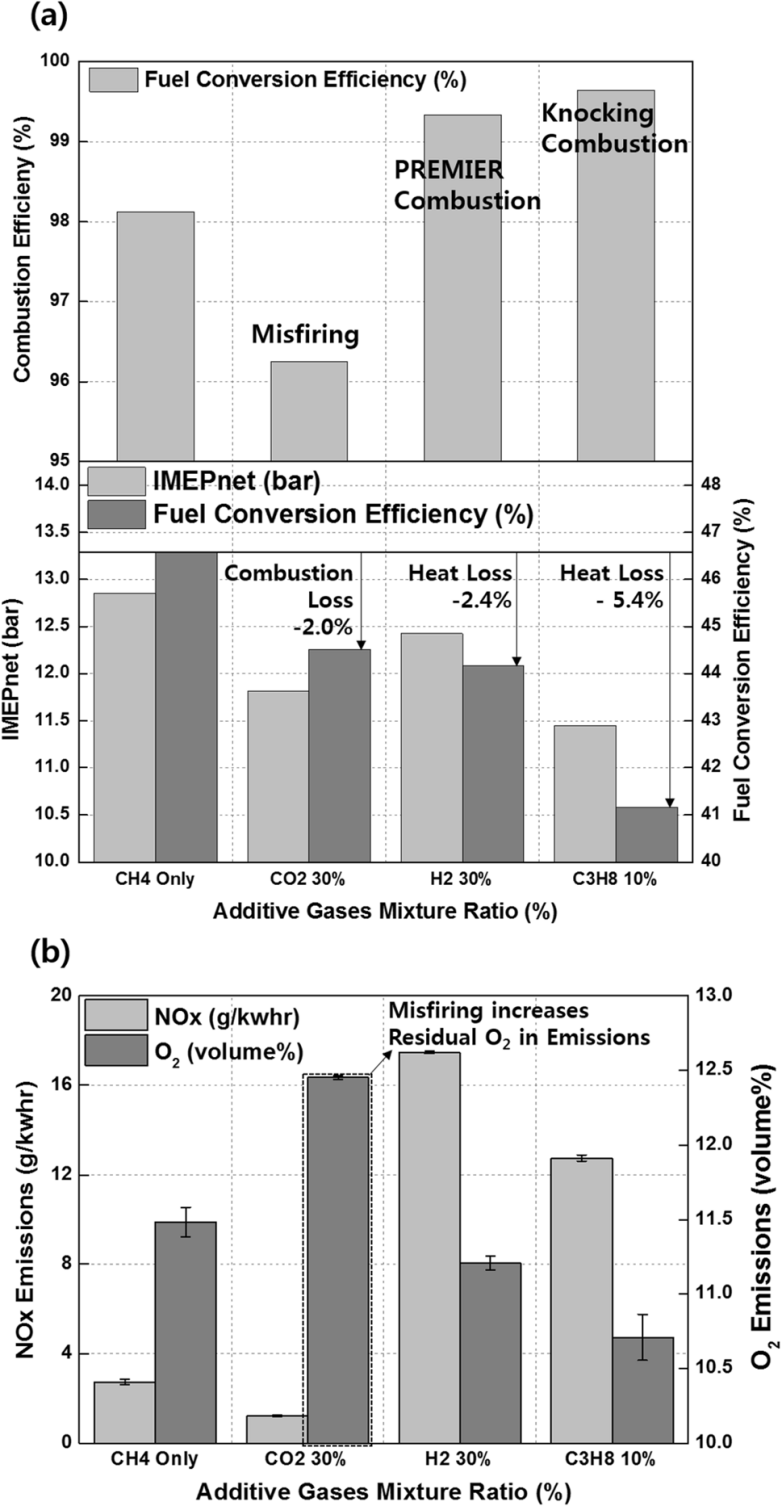
Effects of premixed gas compositions on the (**a**) combustion efficiency, IMEPnet, and fuel conversion efficiency and (**b**) NOx and CO_2_ emissions.

**Figure 22:**
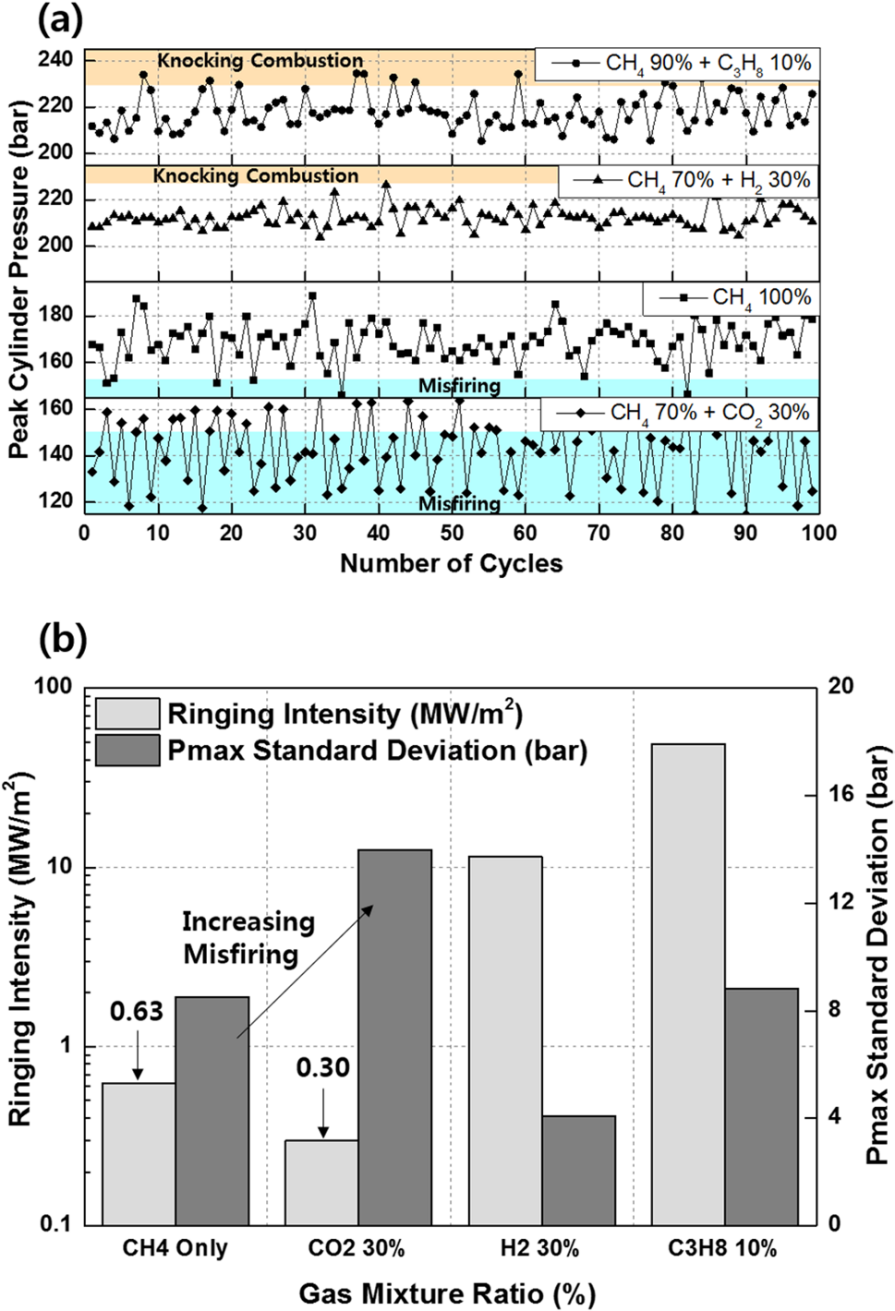
Effects of the premixed gas compositions on the (**a**) cycle to cycle variation in the peak cylinder pressure and (**b**) RI and STD of the peak cylinder pressure.

The effects of CO_2_ on the combustion duration is shown in Fig. 20, in terms of the variation in the MFB with premixed gas compositions. As described previously, C_3_H_8_ and H_2_ gases advance the combustion phasing and decrease the main combustion periods by promoting MPDF combustion. CO_2_ does not considerably affect the ignition delay and initial main combustion periods. After MFB CA 20, when auto-ignition occurs in the end gas region, CO_2_ increases the combustion periods, resulting in the retardation of the end of main combustion at MFB CA 70. This result demonstrates that CO_2_ suppresses the auto-ignition in the end-gas region by decreasing the temperature in the combustion chamber. The effects of CO_2_ on the MPDF combustion in terms of efficiencies and emissions is shown in Fig. 21a and b. Specifically, the effects of the additive gases on the combustion and fuel conversion efficiencies are shown in Fig. 21a. Although the pure CH_4_ conditions correspond to a lower combustion efficiency than that in the case of C_3_H_8_ and H_2_ mixture gases, the highest fuel conversion efficiency is achieved because of the smaller heat losses and negative work. The fuel conversion efficiencies for the H_2_ 30% and C_3_H_8_ 10% cases are 2.4% and 5.4% smaller than those of the pure CH_4_ case, respectively. The presence of H_2_ and C_3_H_8_ gases decreases the fuel conversion efficiency, and the presence of CO_2_ causes misfiring, which decreases the combustion efficiency. Therefore, the fuel conversion efficiency when the CO_2_ mixture ratio is 30% is 2.0% lower than that for pure CH_4_. The NOx and O_2_ emissions shown in Fig. 21b support the explanations of these observations. Because this analysis was focused on CO_2_ as an additive gas, the CO_2_ emissions were not considered. Instead, the O_2_ concentration in the emission gases was compared for investigating the effects of CO_2_ on the MPDF combustion. In the cases of H_2_ and C_3_H_8_ gases, higher NOx emissions are observed than those in the case of pure CH_4_. Moreover, the incomplete combustion products are oxidized, resulting in lower O_2_ concentrations in the emission gases. In contrast, CO_2_ decreases the combustion chamber temperature. The 30% mixture ratio of CO_2_ corresponds to the lowest NOx emissions. In addition, the amount of O_2_ residual gas in the emissions is increased owing to misfiring in the case of high CO_2_ mixture ratios. Both misfiring and knocking combustion increase the combustion variation. As shown in Fig. 22a, when the CO_2_ mixture ratio is 30%, the peak cylinder pressure is significantly decreased owing to misfiring, and peak cylinder pressure exhibits the highest fluctuations. This trend is supported by the results shown in Fig. 22b. When the CO_2_ mixture ratio is 30%, the STD of the peak cylinder pressure is the highest. As the CO_2_ mixture ratio increases, the combustion intensity decreases, resulting in decreased RI values.

### Optimization of the engine operating conditions and premixed gas compositions

**Figure 23:**
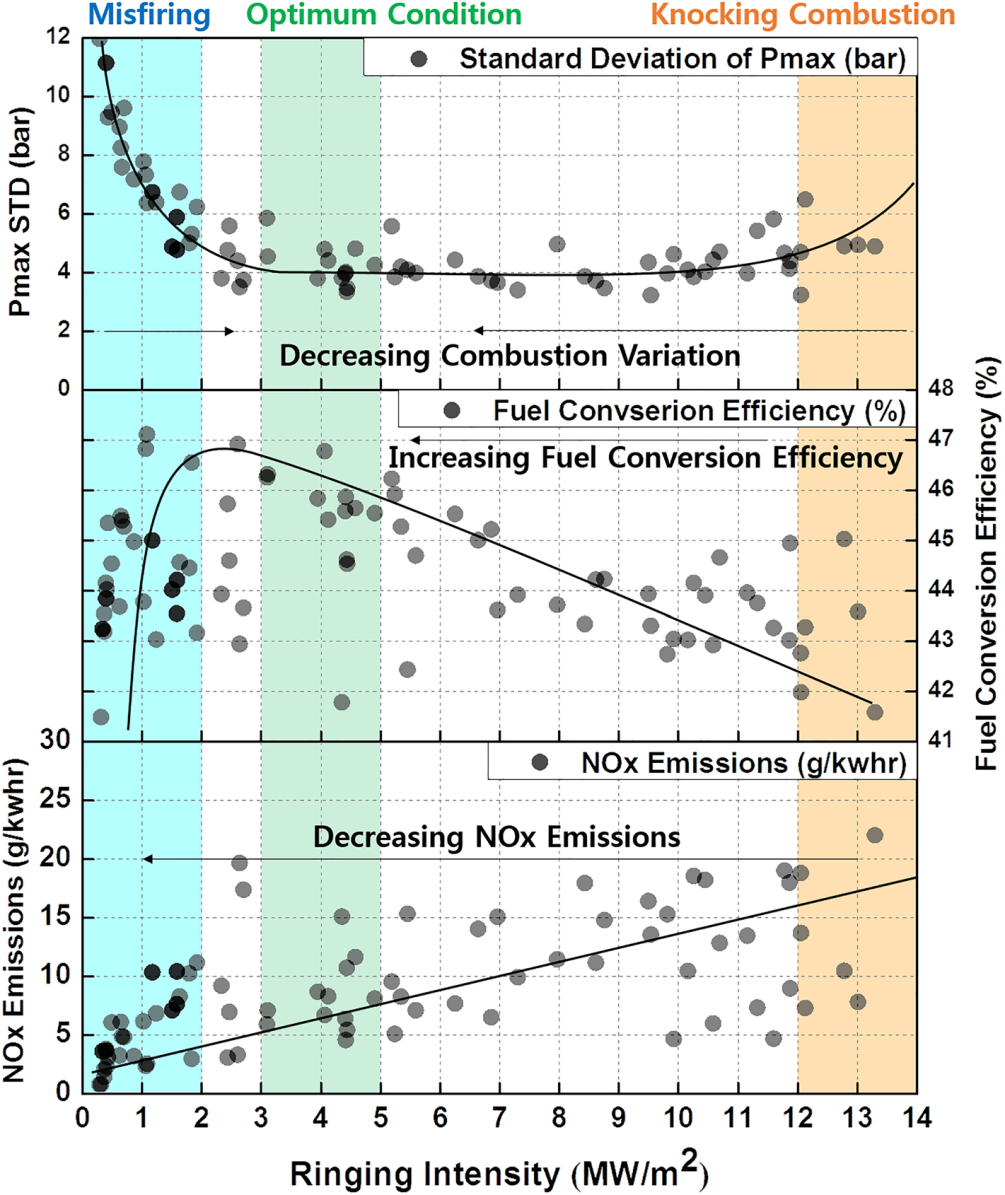
Distributions of the combustion variation, fuel conversion efficiency, and NOx emissions in the middle RI regime.

Before identifying the optimum conditions, the MPDF combustion forms (misfiring, knocking combustion, and PREMIER combustion) must be quantitatively classified in terms of the RI and combustion variation. Therefore, additional experiments were performed for the same premixed gas compositions under various MP injection timings, equivalence ratios, and intake air temperatures. Figure 23 presents the distributions of the experimental results in terms of the RI and engine performance factors. The misfiring, PREMIER combustion, and knocking combustion regimes can be identified in terms of the RI. The low RI regime corresponds to misfiring, with a high combustion variations. The mid-RI regime corresponds to PREMIER combustion, with low combustion variations. The high RI corresponds to knocking combustion, with high combustion variations caused by significantly high peak cylinder pressures. Among the three regimes, the mid-RI regime, corresponding to PREMIER combustion, exhibited the lowest combustion variation. Therefore, this regime was considered as a prerequisite for optimal conditions, and the fuel conversion efficiency and NOx emissions were examined. The fuel conversion efficiency was the highest at the boundary between the low and middle RI regimes. Moreover, the NOx emissions were proportional to the RI. Thus, the engine operating conditions with RI values between 3 MW/m^2^ and 5 MW/m^2^ were considered the optimum conditions.

Various premixed gas compositions and engine operating conditions can satisfy this condition. The engine operating conditions optimized through the addition of C_3_H_8_, H_2_, and CO_2_ gases are summarized in Table 4. Although only a small amount of C_3_H_8_ was added, the engine performance was enhanced under misfiring conditions. Therefore, under low engine operating loads, the engine performance can be increased by adding a small amount of C_3_H_8_ to the gaseous fuel. The effects of H_2_ gas was similar to that of C_3_H_8_, but a large amount of H_2_ was required to achieve the optimum condition owing to its lowest density. The installation of an additional H_2_ storage tank and supply system may be challenging. CO_2_ effectively suppressed knocking combustion by decreasing the combustion temperature. The most convenient technique to increase the CO_2_ concentration in the combustion chamber is to increase the EGR ratio by installing an EGR system.

**Table 4:** Optimum engine operating conditions, obtained by adding various additive gases.

Additive Gas	C_3_H_8_	H_2_	CO_2_
Intake air temperature (°C)	35	35	55
Lambda (1/Equivalence ratio)	2.3	2.2	2.1
MP Inj. timing (bTDC)	27	27	27
Gas compositions	CH_4_ 97% + C_3_H_8_ 3%	CH_4_ 90% + H_2_ 10%	CH_4_ 80% + CO_2_ 20%

## Conclusions

The effects of premixed gas compositions on the MPDF combustion characteristics were analyzed. With CH_4_ gas used as the main power source, C_3_H_8_, H_2_, and CO_2_ gases were added, and engine experiments were conducted. The following conclusions were derived.

1. Because C_3_H_8_ has a low OCN, auto-ignition may easily occur from the local high-temperature region in the combustion chamber at high C_3_H_8_ mixture ratios. This condition leads to knocking combustion accompanied with severe engine vibrations. Knocking combustion significantly decreases the main combustion duration and increases the RI and cycle to cycle combustion variation. Therefore, the fuel conversion efficiency decreases and NOx emissions increase.
2. H_2_, which a high OCN, suppresses knocking combustion. Moreover, H_2_ decreases the ignition delay and initial combustion duration because of the high flame propagation speed. Even at high mixture ratios of H_2_, knocking combustion does not occur. Therefore, the RI and cycle to cycle combustion variation are low. A higher H_2_ mixture ratio corresponds to a lower fuel conversion efficiency because of larger negative work and heat losses. In addition, the advancement in the combustion phasing caused by H_2_ increases the NOx emissions.
3. CO_2_ does not participate in the combustion reaction and has a high heat capacity. Therefore, CO_2_ suppresses MPDF combustion. Auto-ignition does not occur in the end gas region when high CO_2_ mixture ratios are used. Therefore, the main combustion duration increases instead of ignition delay, and misfiring occurs. Although the RI decreases owing to misfiring, the cycle to cycle variation increase. CO_2_ decreases the combustion efficiency and fuel conversion efficiency. Similar to the effect of increasing the EGR rate, increasing the CO_2_ mixture ratio decreases the NOx emissions.
4. The results of this study can be divided into three regimes in terms of the RI value. The low, middle, and high regimes represent misfiring, PREMIER combustion, and knocking combustion, respectively. The middle RI regime corresponds to the lowest combustion variation. In addition to the combustion variation, the fuel conversion efficiency and NOx emissions can be considered to identify the optimum conditions. The optimum conditions correspond to RI values of 3–5 MW/m^2^. The addition of even a small amount of C_3_H_8_ can help enhance the engine performance under misfiring conditions. The effects of H_2_ is the same as that of C_3_H_8_, but a large amount of gas is required owing to its lowest density. Finally, CO_2_ can effectively stabilize MPDF combustion by suppressing the knocking combustion.

## Data Availability

The data that support the findings (experimental results) of this study are available from the corresponding author upon reasonable request.

## Abbreviations

AFR: Air–fuel ratio
aTDC: After top dead center
bTDC: Before top dead center
CA: Crank angle
CI: Compressed ignition
CO: Carbon monoxide
CO_2_: Carbon dioxide
CH_4_: Methane
C_3_H_8_: Propane
C_4_H_10_: Butane
EGR: Exhaust gas recirculation
IMEPnet: Net indicated mean effective pressure
LHV: Lower heating value
MFC: Mass flow controller
MFB: Mass fraction burned
MFM: Mass flow meter
MP: Micro pilot
MPDF: Micro pilot dual fuel
NOx: Nitrogen oxide
O_2_: Oxygen
OCN: Octane number
PM: Particulate matter
PREMIER: Premixed mixture ignition in the end-gas region
RI: Ringing intensity
STD: Standard deviation
TDC: Top dead center
